# Evaluation of Density Functionals for Si–O–C–H Molecule Thermochemistry

**DOI:** 10.1021/acs.jpca.5c04844

**Published:** 2025-10-22

**Authors:** Ingeborg-Helene Svenum, Francesca Lønstad Bleken, Stefan Andersson

**Affiliations:** † 275243 SINTEF Industry, P.O. Box 4760, Torgarden, Trondheim 7465, Norway; ‡ 275243 SINTEF Industry, P.O. Box 124, Blindern, Oslo 0314, Norway

## Abstract

The energies and vibrational frequencies of molecular species with Si–O–C–H compositions have been calculated at the CCSD­(T) level (coupled cluster with single and double excitations and a perturbative treatment of triple excitations). The CCSD­(T) results compare well with experimental data where the difference in enthalpy of formation is typically only about 1–2 kJ/mol in most cases. In addition, the same molecules have been calculated with density functional theory (DFT) calculations using nine commonly used density functionals and two different basis sets. The performance of the DFT calculations is compared with the CCSD­(T) benchmark values in terms of enthalpy of formation, reaction energy, vibrational frequencies, and zero-point energies. The results show that the M06-2X functional provides the lowest mean absolute error (MAE) in terms of the enthalpy of formation, whereas, for the vibrational frequencies and zero-point energies, the SCAN functional gives the lowest MAE values. The results were also grouped according to the types of bonds that are present in the molecules. Moreover, an elaborate set of possible reactions within the molecular species in the Si–O–C–H system is included to evaluate the performance of the different DFT functionals with respect to the relative stability of species within the same reaction system. In this case, the B2GP-PLYP functional shows the smallest errors. PW6B95 is the functional that most consistently performs well for the studied properties of the included molecules. The coupled cluster data sets provide new benchmark data, several of which are not previously available for silicon chemistry.

## Graphical abstract

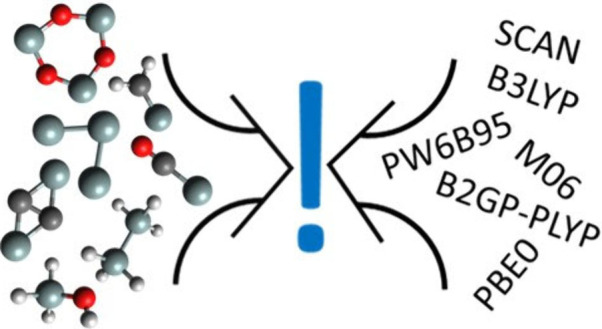

## Introduction

Studies of silicon-containing molecules and their reactions are important for many scientific and technological areas, e.g., preparation of silicon as semiconductor material, combustion of silicon compounds,^1–7^ exhaust from silicon smelters,^8–10^ and the chemistry of silicate dust formation and destruction in circumstellar and interstellar environments.^11–27^ In recent years new techniques for fossil-free production of silicon have been proposed and are under active study.^28^ Since molecular hydrogen is one of the main alternatives to fossil carbon as a reducing agent for the raw material, quartz, this has led to increased interest in understanding reactions in systems containing silicon, oxygen, and hydrogen.

A significant number of computational studies have targeted the reaction mechanisms and thermochemistry of silicon compounds in the gas phase. Different types of density functional theory (DFT) calculations have been used as well as Møller–Plesset perturbation theory (MP2, MP4), coupled cluster techniques and compound methods such as G3 and CBS-Q. For example, Darling and Schlegel^29^ and Allendorf et al.^30^ calculated the enthalpies of formation of SiH_*n*_O and SiH_*n*_O_2_, species relevant for silane combustion. The gas phase reactions of SiO with H_2_,^31–34^ O_2_,^35^ H_2_O,^31,33,35–39^ and OH^33,35,37,38,40^ have been studied by several groups using both experimental and theoretical approaches. The reaction of SiO and H_2_ can proceed through several intermediate steps, initially forming H_2_SiO or cis-HSiOH by breaking the H–H bond of H_2_.^33,34^ These species can be transformed into trans-HSiOH before forming a Si–OH_2_ type species, which is the intermediate step associated with the highest energy, that can split into Si and H_2_O in the gas phase. This reaction is highly endothermic, and the reaction pathway involves several high barriers; therefore, in its basic form, the reaction SiO + H_2_ → Si + H_2_O in the gas phase would be difficult to realize in practice. Under certain conditions SiO can form clusters^26,27^ that will have different reactivity toward other molecules than the parent molecule,^33,37,38^ so given the right conditions reaction with H_2_ might still be possible. Other Si–H-type species can also be considered depending on the SiO and H_2_ balance. On the other hand, reaction of SiO with O_2_, H_2_O or OH lead to formation of SiO_2_.

In a recent study we evaluated reaction mechanisms for the gas-phase reactions of SiO and Si_2_O_2_ with OH and H_2_O.^37^ Based on benchmark coupled cluster (CCSD­(T)) data it was found that the M06 density functional^41^ gave remarkably accurate results for both reaction energies, as well as relative energies between intermediates and barrier heights on the potential energy surfaces for the reactions of SiO with OH and H_2_O. Based on these results it was concluded that M06 would be a suitable choice for the study of reactions involving Si–O–H species, in cases where extremely accurate but computationally demanding methods, such as CCSD­(T), are not practically applicable. However, a more systematic study comparing several different density functionals for many different species and reactions would be helpful in choosing the right approach for the study of complex gas-phase silicon chemistry.

Since traditional silicon production involves carbon as a reductant it would be natural to also study reactions of carbon-containing species with SiO. Nguyen et al.^31^ studied the reaction of SiO with CH_4_ using DFT and CCSD­(T) calculations. All possible reactions were found to be endothermic and to have high barriers. Several Si–C–H and Si–C–O–H species were found as intermediates along the reaction pathways. Considering the interest in small Si–C–O–H species as intermediates and products in different types of reactions, it would be beneficial to also evaluate the accuracy of various DFT methods for the study of such systems.

There have been a significant number of other studies on benchmarking of density functionals on molecular systems,^41–49^ sometimes coupled to development of density functionals with high accuracy for either specific test sets or for consistently good performance for a wide range of molecules and properties. Some of the more specific cases have included transition metal compounds^50–52^ that generally have proven challenging for electronic structure methods. To the best of our knowledge, there have not been any similar systematic studies specifically targeting silicon compounds.

In general, silicon compounds have not featured prominently among databases used to develop density functionals, where functionals are parametrized by fitting to empirical data. The Minnesota functionals, e.g., M06,^41^ M06-2X,^41^ and M11,^44^ as well as B3LYP^49,53^ did have atomization energies of silicon molecules [SiH_2_ (singlet, triplet), SiH_3_, SiH_4_, Si_2_H_6_, Si_2_, SiO, (*only Minnesota:* SiCl_4_, SiF_4_)] the ionization potential (and for *Minnesota* also electron affinity) and the total energy of the Si atom, as well as the proton affinity of SiH_4_ in the training sets used for parametrization. The Minnesota functionals were fitted to several other properties such as barrier heights, noncovalent interactions, and bond dissociation energies, none of which contained any additional silicon species. Functionals originating in the Perdew group, e.g., PBE^54,55^ and SCAN,^56^ were developed to meet exact theoretical constraints and were not fitted to any empirical data. SCAN has for instance been shown to perform exceptionally well for calculating structures and energetics of a wide range of oxide polymorphs, including SiO_2_, in the solid phase.^57^ It is therefore of interest to compare the different classes of density functionals and assess whether training on silicon compounds makes a significant difference in the performance for describing other silicon species or not.

In this study, we not only focus on the overall performance of density functionals for the energetics of silicon compounds and related species but also focus on the performance for specific bond types, both energies as well as vibrational frequencies and zero-point energies. In the process we have created benchmark data sets for the different properties studied. We hope that this will be a valuable guide for future studies targeting specific types of silicon molecules and also as a basis for elementary steps in various silicon reaction chemistries.

## Computational Details

Benchmark energies were calculated with CCSD­(T) (coupled cluster with single and double excitations and a perturbative treatment of triple excitations). For standard frozen core calculations, the basis sets aug-cc-pV­(X+d)­Z (X = T, Q, 5, 6) were used. To treat core–valence (CV) correlation effects, calculations were run with and without all core electrons (except 1s on Si) included in the correlation calculations using the cc-pwCVXZ (X = T, Q, 5) basis sets. In both cases energies were extrapolated to the complete basis set (CBS) limit using the extrapolation formula^58,59^ *E*(CBS) = *E*(*l*_max_) + *A*/(*l*_max_ + 1/2)^4^, where *l*_max_ is the highest angular momentum value in the basis set. Scalar relativistic corrections were added by running CCSD­(T) calculations with and without the DPT2 Hamiltonian^60–62^ with an uncontracted cc-pVTZ basis set. Corrections for spin–orbit energies of the fine-structure states of C, O and Si were added. For most species, geometry optimization and vibrational frequency calculations were performed using CCSD­(T)/aug-cc-pV­(Q+d)­Z calculations, but for SiH_3_SiH_3_ and SiH_3_SiH the aug-cc-pV­(T+d)­Z basis set was used instead. In the cases of Si_2_O_4_ and Si_3_O_3_, geometries were calculated using both aug-cc-pV­(T+d)­Z and aug-cc-pV­(Q+d)­Z. The aug-cc-pV­(Q+d)­Z geometry was then used for calculating the electronic energy, whereas the aug-cc-pV­(T+d)­Z geometry was used for frequency calculations at the CCSD­(T)/aug-cc-pV­(T+d)­Z level. The CCSD­(T) calculations were extrapolated to the CBS limit using calculations employing three different basis sets, as mentioned above. In most cases the frozen core calculations were extrapolated using aug-cc-pV­(X+d)­Z basis sets with X = Q, 5, 6, but for Si_2_O_3_, Si_2_O_4_, Si_3_O_3_, linear Si_2_C_2_, SiC_3_, SiH_3_SiH_3_, SiH_3_SiH, C_2_H_2_, C_2_H_4_, and C_2_H_6_ the X = T, Q, 5 basis sets were used instead. The CFOUR package was used for the CCSD­(T) calculations.^63^

Geometry optimizations were performed and vibrational frequencies were calculated with Density Functional Theory (DFT) for all molecules with the NWChem computational software package version 7.0.0.^64–66^ The following functionals were used: B2GP-PLYP,^67^ B3LYP,^49,53^ M06,^41^ M06-2X,^43^ M11,^44^ PBE0,^68^ PBE,^54,55^ PW6B95,^69^ and SCAN,^56^ as listed in Table 1. The spherical minimally augmented correlation-consistent polarized Valence Triple Zeta basis set with additional diffuse functions, maug-cc-p­(T+d)­Z (hereinafter called TZ), as well as the maug-cc-pV­(Q+d)­Z (hereinafter called QZ) basis set were employed.^70–72^ The total energy was converged to 10^–7^ and the RMS of the density matrix was converged to 10^–6^. Grid and tolerances were specified to “huge” and “tight” according to NWChem predefined settings, respectively. Only positive vibrational frequencies gave confirmation that a true minimum was obtained. For some linear molecules, small imaginary frequencies were obtained. These are due to numerical instabilities and do not reflect the correct physical system. However, they are included in the analysis. Finally, C_2_ is only included in the CCSD­(T) calculations and is not reported in the DFT results. It is known in literature that molecular C_2_ is particularly challenging and has been described as being a prototypical example of multireference character in electronic structure.^73^ Repeated attempts provided several electronic states for C_2_ but not the singlet ground state. See the Supporting Information for an extended discussion on the issues of C_2_.

**Table 1:** Summary of the Different Theoretical Methods Used in This Study

**Method**	**Package**	**Functional**	**Type**
Coupled cluster	CFOUR		
DFT	NWChem	PBE	GGA
		PBE0	hybrid
		B3LYP	hybrid
		B2GP-PLYP	double hybrid
		PW6B95	hybrid meta-GGA
		M06	hybrid meta-GGA
		M06-2X	hybrid meta-GGA
		M11	range-separated hybrid meta-GGA
		SCAN	meta-GGA

In order to estimate whether any of the included species were not sufficiently well-described by CCSD­(T) calculations, the %TAE­[(T)] diagnostic^74,75^ was evaluated: %TAE[(T)] = 100 × TAE[CCSD(T)] – TAE[CCSD]/TAE[CCSD(T)]. Here TAE is the total atomization energy of a molecule at the CCSD­(T) and CCSD level using the largest basis set employed. The %TAE­[(T)] diagnostic has been shown to be useful to estimate whether CCSD­(T) covers a sufficient amount of the correlation energy, i.e, only minor nondynamical correlation contributions, or whether post-CCSD­(T) calculations would be necessary for calculated energies to be of benchmark quality. Typically, a %TAE­[(T)] below 5% indicates post-CCSD­(T) contributions less than 0.5 kcal/mol (2 kJ/mol), below 10% the post-CCSD­(T) contributions are less than 1 kcal/mol (4 kJ/mol), but for %TAE­[(T)] larger than 10% they could contribute significantly more than 1 kcal/mol. In the latter case, CCSD­(T) calculations would be considered to have too high uncertainties to be safe to use as a benchmark. In Table S1 the %TAE­[(T)] for the studied species are shown. It is only the singlet C_2_ molecule that has a value above 10% (at 13.4%). Interestingly enough, the CCSD­(T) results on singlet C_2_ were quite reasonable in comparison to experiments (see Table 2 and Table S2), but the DFT calculations invariably failed to give results in qualitative agreement with accurate values. Other molecules apart from singlet C_2_ to have relatively large %TAE­[(T)] are Si_3_ (8.6%), O_2_ (7.0%), triplet C_2_ (6.4%), SiC (6.0%), CSiO (5.9%), Si_2_ (5.8%), C_3_ (5.6%), CSi_2_ (5.2%), and linear Si_2_C_2_ (5.2%). All of these were retained in the data set, and only C_2_ was excluded from the evaluation of density functionals.

**Table 2:** Benchmark Data from CCSD­(T) Calculations: Multiplicity (*M*), Zero-Point Energies (*E*_zpe_), and Calculated and Experimental Enthalpy of Formation Evaluated at 0 K (Δ*H*_f,0_^**0**^) and 298.15 K (Δ*H*_f,298_^0^)­^a^

**Species**	***M***	***E***_**ZPE**_ (kJ/mol)	Δ ***H***_**f,0**_^**0**^ (kJ/mol)	Δ ***H***_**f,298**_^**0**^ (kJ/mol)	Δ ***H***_**f,298**_^**0**^ **(exp)** **(kJ/mol)**
C	3	0.0	711.2	716.7	716.67 ± 0.46,^b^ 716.889 ± 0.044^e^
Si	3	0.0	448.3	452.7	452.7 ± 0.6,^c^ 454.70 ± 0.59^e^
O	3	0.0	246.4 (*245.1*)	248.2 (*247.5*)	249.17 ± 0.10,^b^ 249.229 ± 0.0021^e^
O_2_	3	9.5	0.0	0.0	0.0
CO	1	12.9	–115.2 (*−114.6*)	–111.6 (*−111.3*)	–110.53 ± 0.17,^b^ –110.524 ± 0.026^e^
CO_2_	1	30.4	–396.5 (−394.9)	–396.6 (*−395.3*)	–393.522 ± 0.05,^b^ –393.476 ± 0.015^e^
C_2_	1	11.1	821.6 (*823.1*)	828.9 (*829.7*)	837.7 ± 3.8,^b^ 830.46 ± 10,^d^ 828.47 ± 0.092,^e^ *826.577 ± 0.092* ^e^ *(singlet only)*
C_2_	3	9.8	830.4 (*832.9*)	837.7 (*839.6*)	*833.933 ± 0.092* ^e^ *(triplet only)*
C_3_	1	20.8	815.1 (*816.8*)	825.8 (*826.5*)	820 ± 17,^b^ 823.5 ± 0.53^e^
SiO	1	7.4	–104.2 (*−101.5*)	–101.7 (*−101.9*)^a^	–100.42 ± 8.4,^b^ –97.2 ± 0.54^e^
SiO_2_	1	17.9	–290.1 (*−285.1*)	–289.6 (*−286.1*)^a^	–305.43 ± 33.5,^b^ –322.07 ± 10,^d^ –279.0 ± 2.0^e^
Si_2_	3	3.1	581.4 (*586.1*)	586.9 (*588.6*)	589.9 ± 13,^b^ 583.86 ± 22,^d^ 591.4 ± 1.6^e^
Si_3_	1	7.6	624.0 (631.8)	630.9 (*634.2*)	636.0 ± 42^b^
Si_2_O	1	11.4	112.1 (*117.7*)	115.2 (*117.8*)	-
Si_2_O_2_	1	22.9	–435.0 (*−427.8*)	–434.6 (*−430.5*)	–465.3 ± 42^f^ (Δ*H*_f,0_^0^), –410.0 ± 33.5^g^
SiC	3	5.9	742.7 (*745.7*)	748.9 (*750.1*)	719.6 ± 33,^b^ 745.3 ± 2.4^e^
SiC_2_	1	16.5	632.4 (*636.5*)	640.8 (*642.7*)	615.0 ± 29^b^
CSi_2_	1	13.2	548.1 (*554.1*)	555.6 (*558.1*)	535.6 ± 25^b^
Si_2_C_2_	1	24.8	686.0 (*693.2*)	694.0 (*697.4*)	-
SiCO	3	19.2	200.2 (*203.7*)	204.0 (*206.1*)	-
CSiO	3	12.1	470.7 (*474.9*)	477.2 (*479.5*)	-
H	2	0.0	215.9 (*215.9*)	217.8 (*217.8*)^a^	217.999 ± 0.006,^b^ 217.998 ± 0.000,^e^ 217.998 ± 0.006^h^
H_2_	1	26.3	0.0	0.0	0.0
OH	2	22.4	36.5 (*35.8*)	36.5 (*36.4*)^a^	38.987 ± 1.21,^b^ 37.522 ± 0.025^e^
H_2_O	1	56.4	–240.1 (*−239.3*)	–243.2 (*−242.5*)^a^	–241.826 ± 0.042,^b^ –241.801 ± 0.025^e^
CH	2	17.1	592.6 (*593.0*)	596.2 (*596.4*)	594.128 ± 17.5,^b^ 596.171 ± 0.097^e^
CH_2_	3	45.5	390.7 (*391.7*)	391.3 (*391.9*)	386.39 ± 4.2,^b^ 391.6 ± 0.096^e^
CH_3_	2	78.2	149.3 (*150.4*)	146.1 (*146.9*)	145.687 ± 0.8,^b^ 146.471 ± 0.053^e^
CH_4_	1	117.6	–66.3 (*−65.1*)	–74.3 (*−73.5*)	–74.873 ± 0.34,^b^ –74.520 ± 0.048^e^
SiH	2	12.2	368.0 (*371.8*)	370.5 (*373.2*)	376.66 ± 8.4,^b^ 368.64 ± 8.00,^d^ 373.11 ± 0.76^e^
SiH_2_	1	30.9	267.7 (*270.6*)	267.2 (*268.6*)	273.33 ± 5.00,^d^ 269.77 ± 0.86,^e^ 288.7 ± 8.4^i^
SiH_3_	2	56.2	198.8 (*203.3*)	194.3 (*197.3*)	200.50 ± 2.50,^d^ 199.32 ± 0.86,^e^ 202.9 ± 6.7^i^
SiH_4_	1	82.5	36.2 (*41.1*)	27.5 (*30.9*)	34.31 ± 2.1,^b^ 34.70 ± 1.50,^d^ 33.13 ± 0.66^e^
H_2_SiC	1	41.4	675.3 (*680.2*)	676.4 (*679.4*)	-
H_2_CSi	1	57.0	321.3 (*324.9*)	321.5 (*323.3*)	-
HCSiH	1	50.1	465.5 (*470.3*)	465.6 (*468.6*)	-
HOSi	2	32.9	–8.0 (*−4.6*)	–8.2 (*−6.4)*	-
HSiO	2	22.7	24.5 (*28.5*)	24.3 (*26.8*)	-
H_2_SiO	1	48.6	–103.1 (*−98.2*)	–107.3 (*−103.9*)	-
cis-HSiOH	1	54.1	–99.2 (*−95.4*)	–103.2 (*−101.0*)	-
trans-HSiOH	1	55.1	–98.8 (*−94.2*)	–103.0 (*−100.6*)	-
H_2_CO	1	70.0	–107.2 (*−105.8*)	–110.9 (*−109.8*)	–115.897 ± 6.3,^b^ –108.70 ± 0.50,^d^ –109.229 ± 0.096^e^
HCO	2	34.2	40.3 (*41.4*)	40.9 (*41.7*)	43.5 ± 8,^b^ 42.00 ± 5.00,^d^ 41.755 ± 0.096^e^
H_3_SiOH	1	101.4	–280.3 (*−273.8*)	–291.2 (*−286.7*)	-
Si_2_O_3_	1	35.2	–743.1 (*−732.2*)	–744.9 (*−737.0*)	-
Si_2_O_4_	1	46.6	–1002.8 (*−988.8*)	–1006.5 (−*995.4*)	-
Si_3_O_3_	1	37.5	–789.5 (*−776.6*)	–788.8 (*−780.4*)	-
Si_2_C_2__linear	1	23.9	774.1 (*783.8*)	784.3 (*790.2*)	-
SiC_3__linear	1	27.6	921.0 (*927.7*)	932.2 (*936.3*)	-
C_2_H_2_	1	69.4	226.0 (*229.2*)	225.9 (*228.4*)	226.731 ± 0.79,^b^ 227.4 ± 0.8^d^
C_2_H_4_	1	133.4	57.8 (*61.5*)	49.5 (*52.5*)	52.467 ± 0.29,^b^ 52.4 ± 0.5^d^
C_2_H_6_	1	196.2	–71.2 (*−67.3*)	–87.0 (*−83.8*)	–84.0 ± 0.4^j^
SiH_3_SiH_3_	1	128.8	76.6 (*88.8*)	62.6 (*71.7*)	80.30 ± 1.5^k^
SiH_3_SiH	1	80.2	306.3 (*316.0*)	300.2 (*307.0*)	-

a Numbers in parentheses for Δ*H*_f,0_^0^ and Δ*H*_f,298_^0^ include relativistic corrections (scalar and spin-orbit; see text for details).

b Andersson.^37^

c NIST-JANAF.^76^

d Karton and Martin.^77^

e Gurvich et al.^78^

f ATcT.^79^

g Wu et al.^80^

h Zmbov et al.^81^

i Cox et al.^82^

j Boo et al.^83^

k Manion.^84^

l Rappoport and Apeloig.^85^

Due to convergence issues, vibrational frequencies calculated using the TZ basis set were used instead of QZ calculations in the final analysis in a few cases: SiC_3__linear calculated with the PBE0 functional and Si_2_O_4_ and SiH_3_SiH calculated with the B3LYP functional. The overall conclusions are not expected to be affected by this.

The reported enthalpies of formation (evaluated at 0 and 298.15 K) are calculated with respect to the elemental constituents in their standard state for O (O_2_) and H (H_2_) or for elemental single atom for Si and C as shown in eq 1. The enthalpies of C and Si are corrected based on the literature values 711.185 (716.670) kJ/mol^76^ for C and 448.316 (452.667) kJ/mol^77^ for Si evaluated at 0 (298.15) K.

1 $${\Delta H}_{f}\left({Si}_{x}O_{y}H_{z}C_{w}\right)=H\left({Si}_{x}O_{y}H_{z}C_{w}\right)-x\;*\;H\left(Si\right)-y\left(\frac{1}{2}H\left(O_{2}\right)\right)-z\left(\frac{1}{2}H\left(H_{2}\right)\right)-w\;*\;H\left(C\right)$$

Mean absolute error (MAE) and mean error (ME) of enthalpy of formation are calculated with the coupled cluster results as a reference. MAE and ME are also calculated for frequencies with each frequency given equal weight (i.e., all frequencies are compared one to one), for which also the mean absolute percentage error (MAPE) was also evaluated. In addition, the MAE and ME for zero-point energies (ZPEs) have been evaluated.

## Results and Discussion

The results for the different atoms and molecules calculated using CCSD­(T) in this study are listed in Table 2, together with the zero-point energy, *E*_ZPE_, and enthalpy of formation at 0 K (Δ*H*_f,0_^0^) and 298.15 K (Δ*H*_f,298_^0^). Experimental values are given for the enthalpy of formation at 298.15 K, if available. The experimental values should be compared to the CCSD­(T) results that include relativistic corrections (numbers in parentheses in Table 2). For the comparison between CCSD­(T) and DFT discussed below, the enthalpies of formation are calculated without relativistic corrections.

The enthalpies of formation calculated with CCSD­(T) are in most cases within chemical accuracy (i.e., 1 kcal/mol, 4 kJ/mol) of available literature values. In some cases, the discrepancy is larger, but this is mainly for lesser studied species with large experimental uncertainties, such as Si_2_O_2_, SiC_2_, and CSi_2_, and species with wide ranges of literature values (SiO_2_, SiC, and SiH_2_). In two cases, the discrepancy is between and 4 and 8 kJ/mol in comparison to a single literature value with small error bars (triplet C_2_ and SiH_3_SiH_3_), but for significantly more species there is only about 1 kJ/mol difference between calculated and literature values (CO, CO_2_, H, H_2_O, HCO, C_2_H_2_, C_2_H_4_, C_2_H_6_). We conclude that the accuracy of the CCSD­(T) data is sufficiently high to motivate their use as benchmark data.

All of the species listed in Table 2 (except C_2_) were also calculated using DFT with the TZ and QZ basis sets using the selected functionals. Figure 1 shows the mean error and mean absolute error with standard deviations of the Δ *H*_f,0_(Si_*x*_O_*y*_H_*z*_C_*w*_) energy at the DFT/QZ level compared to the CCSD­(T) results. For a more comprehensive figure including Δ*E*_f,0_(Si_*x*_O_*y*_H_*z*_C_*w*_) and Δ *H*_f,298_(Si_*x*_O_*y*_H_*z*_C_*w*_) and the triple-ζ basis set we refer to Figure S1 in the Supporting Information. The MAE of the enthalpy for all of the functionals using both basis sets are also listed in Table 3, together with the MAE and mean absolute percentage error (MAPE) of vibrational frequencies and MAE of zero-point energies.

**Table 3:** Mean Absolute Error for Enthalpies of Formation at 0 K (Δ*H*_f,0_^0^) in kJ/mol, Zero-Point Energy in kJ/mol and Frequencies in cm^–1^ and Mean Absolute Percentage Error for Frequencies Compared to CCSD­(T) Calculations for All Species Considered in This Study

			**Frequencies**	
**Method**	**Δ*H***_**f,0**_^**0**^ **(kJ/mol)**	***E***_**zpe**_ **(kJ/mol)**	**(cm^–1^)**	**(%)**
B2GP-PLYP TZ	19.5	0.7	21.8	4.2
B2GP-PLYP QZ	10.9	0.7	23.0	4.0
B3LYP TZ	27.7	0.5	20.3	4.1
B3LYP QZ	23.8	0.4	18.6	3.7
M06 TZ	15.4	0.7	26.1	4.9
M06 QZ	17.0	0.7	26.0	4.7
M06-2X TZ	9.0	0.8	28.0	4.3
M06-2X QZ	8.9	0.8	28.5	4.3
M11 TZ	20.8	0.6	26.0	5.0
M11 QZ	15.8	0.8	33.4	5.7
PBE0 TZ	28.7	0.5	21.0	3.8
PBE0 QZ	26.5	0.5	20.3	4.0
PBE TZ	43.6	1.6	50.6	5.4
PBE QZ	43.5	1.6	48.8	5.2
PW6B95 TZ	14.7	0.5	19.8	3.8
PW6B95 QZ	13.7	0.5	20.1	3.6
SCAN TZ	23.1	0.5	17.7	2.4
SCAN QZ	21.7	0.4	16.5	2.2

**Figure 1:**
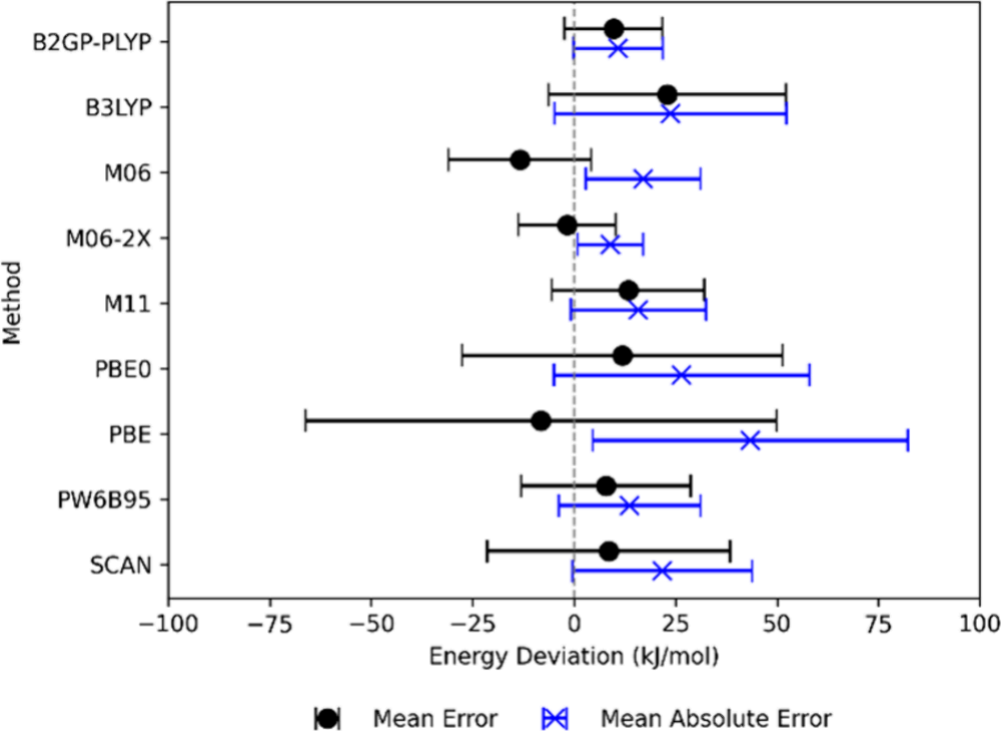
Mean absolute error (blue) and mean error (black) for the enthalpies of formation at 0 K (Δ*H*_f,0_^0^) for selected functionals using the QZ basis set. Mean absolute errors and mean errors are calculated with respect to the coupled cluster results. Standard deviations are included as error bars.

The different functionals give mean errors in energy that can be either positive or negative for the given set of species, as shown in Figure 1. The analysis of density functional performance will in general be based on results for the QZ basis set. The mean absolute error is lowest for the M06-2X functional at 8.9 kJ/mol. The M06-2X functional is followed by B2GP-PLYP < PW6B95 < M11 < M06 < SCAN that have MAEs of 10.9, 13.7, 15.8, 17.0, and 21.7 kJ/mol, respectively. M06-2X has a mean error close to zero since the formation enthalpy is just as likely to be overestimated as underestimated for the set of species studied. M06-2X, B2GP-PLYP, M06 and M11 are the functionals with the smallest standard deviations. The M11 functional overestimates the enthalpies and there is thus only a minor difference between the mean absolute error (blue cross and bar) and the mean error (black dot and bar). M06 gives a mean error below zero, meaning that the enthalpy of formation is more likely to be underestimated compared to the coupled cluster results. The same is true for the PBE functional. However, the PBE results have a much larger spread, and the formation energy can be underestimated or overestimated for some of the species involved (see Figure S1 in the Supporting Information). As a consequence, the mean absolute error is large (43.5 kJ/mol), even though the mean error (−8.1 kJ/mol) does not deviate much from 0, and the standard deviation is also largest for PBE. The other nonhybrid functional, SCAN, has a MAE close to that of the Minnesota functionals (M06, M06-2X and M11) with a rather small mean error of 8.5 kJ/mol and a significantly smaller standard deviation than PBE. B3LYP and PBE0, which are both hybrid functionals, perform worse than the Minnesota functionals with MAEs of 23.8 and 26.5 kJ/mol, respectively. However, B3LYP generally overestimates the formation enthalpy for the species investigated, whereas PBE0 errors are more evenly spread around zero. There is no strong basis set effect (see Table 3 and Figure S1), and the TZ and QZ basis sets give quite similar results, with the notable exception of the B2GP-PLYP double hybrid functional where the MAE for QZ is almost half of that for TZ.

It would seem that the density functionals trained on silicon data perform better for the enthalpies of formation. However, B3LYP and SCAN have similar MAEs, which means that it is not strictly necessary to train the functionals on similar data to achieve reasonable accuracy for molecular systems. It would also seem that pure meta-GGAs, such as SCAN, can be constructed to achieve the same accuracy for enthalpies of formation as traditional hybrid functionals such as B3LYP and PBE0. The differences between B3LYP and the Minnesota functionals might be attributed to either the latter being more advanced hybrid meta-GGA functionals or to more general molecular data being included in the fitting procedure for the Minnesota functionals since the amount of data on silicon species in the respective training sets is quite similar. In a recent study, the quality of density functionals for predicting the enthalpies of formation of 421 molecular species was reported.^48^ The data set was dominated by organic compounds and did not include any silicon species, thereby being fully complementary to the present study. Their conclusion on density functionals also included in this work (PBE, PBE0, B3LYP, M06, and M06-2X) was that M06-2X showed the best performance and PBE clearly the worst. PBE0, B3LYP and M06 gave results of intermediate quality but not hugely better than PBE, with no clear order between them. This is in reasonable agreement with the results for silicon compounds, and the general quality of the results therefore seems to be more universal than just restricted to one class of compounds.

In Table S2, the 274 harmonic vibrational frequencies (including degenerate vibrations for linear molecules) of all included molecular species as calculated using CCSD­(T) with the QZ basis set are listed alongside the available experimental harmonic and fundamental vibrational frequencies. The CCSD­(T) frequencies are in most cases within 10 cm^–1^ (36 of 44; 82%), whereof 24 (55%) are within 5 cm^–1^ and 11 (25%) are within 2 cm^–1^, of the available experimentally derived harmonic frequencies. The largest deviations are found for vibrations in SiC_2_ (809 vs 844 cm^–1^), CH_4_ (1344 vs 1367 cm^–1^), C_2_H_2_ (603 vs 624 cm^–1^), and SiC (985 vs 965 cm^–1^).

The vibrational frequencies are mostly overestimated for B2GP-PLYP, M06, M06-2X, and PW6B95, both over- and underestimated with B3LYP, M11, PBE0, and SCAN, and mostly underestimated for PBE using CCSD­(T) as a reference, as shown in Figure 2 (upper panel), where the MAE lies in the range 16.5–48.8 cm^–1^. Figure S2 shows a detailed analysis for vibrational frequencies. As for the enthalpies, PBE performs the worst for frequency, with an MAE of 48.8 cm^–1^. In this case, the Minnesota functionals do not perform better than PBE0, B3LYP or SCAN. Instead, the best-performing functional for frequencies is SCAN (MAE: 16.8 cm^–1^), closely followed by B3LYP (18.6 cm^–1^), PW6B95 (20.1 cm^–1^), and PBE0 (20.3 cm^–1^), whereas M06, M06-2X and M11 give mean absolute errors of 26.0, 28.5, and 33.4 cm^–1^, respectively. The mean errors for SCAN and B3LYP (see Figure 2) are close to zero, indicating that these functionals provide the most balanced description of vibrational frequencies, but the SCAN results have smaller standard deviation, indicating that it is the most reliable density functional for vibrational frequencies. Basis set effects on vibrational frequencies calculated by DFT are minor, as the TZ basis set provides MAE values similar (equal or slightly lower) to those of the QZ basis set (see Figure S2 and Table S3).

**Figure 2:**
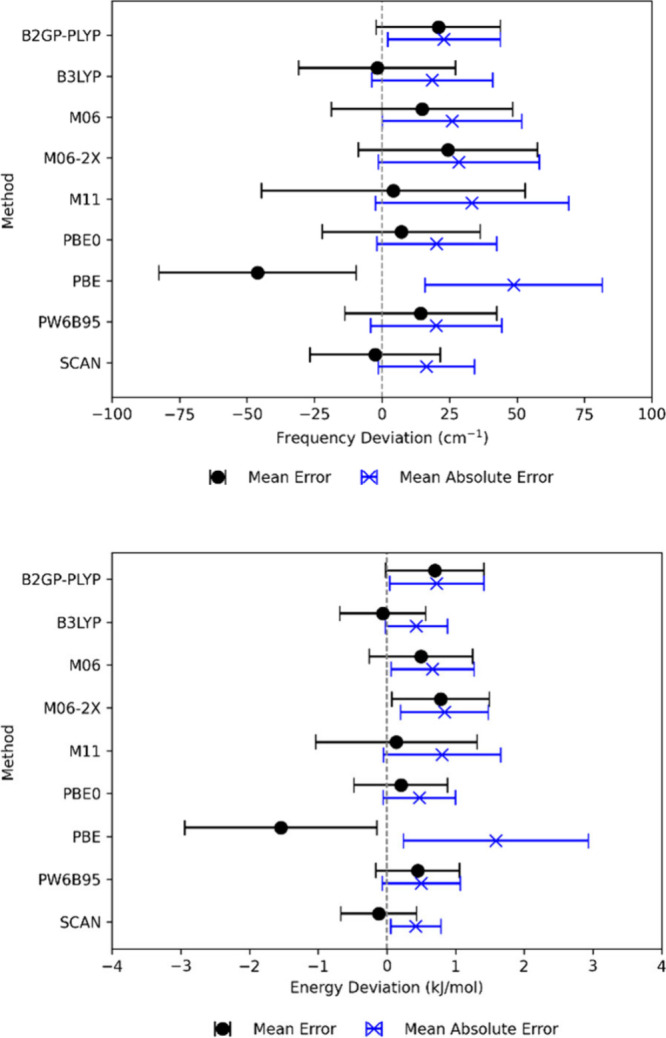
Mean absolute error (blue) and mean error (black) for the calculated (upper panel) frequencies and (lower panel) zero-point energy (ZPE) for selected functionals using the QZ basis set. Errors are calculated with respect to the coupled cluster results. Standard deviations are included as error bars.

In the case of DFT calculations, it has previously been shown that the accuracy of calculated vibrational frequencies depends on the level of theory,^86,87^ where the vibrational frequencies often are overestimated compared to experimental observations.^88^ This is generally the case when applying the simple harmonic oscillator model,^89^ neglecting anharmonicity.^90^ Different scaling procedures have been proposed in order to correct the calculated harmonic vibrational frequencies to better match the measured fundamental frequencies.^46,87,88,91^ Lower frequencies have a greater impact on thermal contributions (enthalpy and entropy) whereas higher frequencies contribute more to zero-point energies.^91^ Different scaling factors for low (<1000 cm^–1^) and high frequencies have been considered in previous studies.^46,88,92^ In the present work, we have not distinguished between high and low frequencies and have also not applied any scaling factors, i.e., only harmonic frequencies are compared. The main aim is to identify density functionals that provide results similar to those from CCSD­(T) calculations. When comparing the calculated MAE in Table 3, the MAE of the frequencies differs depending on functional, but the choice of basis set does not seem to have a large impact. This agrees with previous results comparing various Minnesota functionals.^92^ Zhao and Truhlar^41^ evaluated several density functionals for a set of 38 harmonic frequencies for small molecules. Of the density functionals also considered here (B3LYP, PBE, M06, and M06-2X), B3LYP clearly performed best for vibrational frequencies, whereas PBE, M06, and M06-2X had errors that were approximately double that of B3LYP.

In Figure 2 (lower panel) and Table 3, the results for zero-point energies are shown. As noted above, the ZPE contain a higher weight of contributions from high vibrational frequencies (with high energy) so it is not necessarily the same information as in the comparison of vibrational frequencies. However, the picture does emerge as quite similar to that of the frequencies themselves. Overall, SCAN has the lowest MAE (0.4 kJ/mol) and the smallest spread around the mean energy. B3LYP has the same MAE as SCAN and a similar ME (−0.1 kJ/mol) but has somewhat larger standard deviations of the errors. PBE0 and PW6B95 also have rather small MAE (0.5 kJ/mol). PBE shows the largest errors for ZPE (MAE: 1.6 kJ/mol; ME: −1.5 kJ/mol), as would be expected from its performance for vibrational frequencies. Interestingly enough, Zhao and Truhlar^41^ found that PBE had a relatively small error for ZPE, which was even smaller than B3LYP. This is in contrast with our findings and somewhat surprising considering the general performance of PBE for vibrational frequencies.

In Table 3 we also show the mean absolute percentage error (MAPE) of the vibrational frequencies. The MAPE will be more sensitive to errors in the low frequency range, and, as discussed above, therefore correlates with uncertainties in the calculation of properties dependent on entropy, such as free energies. Here SCAN performs clearly much better than the other functionals with a MAPE of 2.2%, and is followed by PW6B95, B3LYP, PBE0, B2GP-PLYP at 3.6%, 3.7%, 4.0%, and 4.0%, respectively. Something that was not directly apparent from the absolute errors of the frequencies and ZPE, is that M11 shows the largest MAPE (5.7%), indicating worse performance than PBE (5.2%) for parts of the frequency spectrum.

### Bond Types

In Tables 4 and 5 and Tables S3–S5 the results on enthalpies of formation, vibrational frequencies, and zero-point energies have been divided into several categories depending on which types of bonds the molecules contain. The results are weighted per molecule, meaning that species containing one or several bonds of the same type have the same weight. In that way it is possible to predict how the different density functionals are expected to behave for general molecules containing combinations of these types of bonds: Si–Si, O–H, Si–H, Si–C, Si–O, and C–H. Note that we do not distinguish between single, double or triple bonds in the analysis. In general, there are similar trends comparing the bond-resolved results to the overall results discussed above, but there are some remarkable details that become apparent.

**Table 4:** Mean Absolute Errors for Enthalpies of Formation at 0 K (Δ*H*_f,0_^0^) in kJ/mol Compared to CCSD­(T) Calculations According to Species Containing Bonds of Type “Si–Si”, “O–H”, “Si–H”, “Si–C”, “Si–O” or “C–H”

	**MAE of Δ*H***_**f,0K**_^**0**^ (kJ/mol)					
**Method**	**Si–Si**	**O–H**	**Si–H**	**Si–C**	**Si–O**	**C–H**
B2GP-PLYP TZ	39.8	12.5	14.2	25.8	31.2	9.8
B2GP-PLYP QZ	24.8	6.9	7.9	13.4	17.8	5.2
B3LYP TZ	64.1	22.2	20.2	24.0	54.8	11.8
B3LYP QZ	58.5	19.9	17.5	16.9	49.0	10.1
M06 TZ	23.3	9.3	20.3	22.7	12.8	7.6
M06 QZ	24.3	9.3	19.9	28.5	12.2	8.5
M06-2X TZ	10.6	3.9	8.3	14.6	10.6	6.9
M06-2X QZ	8.7	4.4	7.7	15.7	10.8	6.7
M11 TZ	38.8	4.6	14.4	34.6	28.2	12.1
M11 QZ	27.3	5.0	10.2	28.8	18.4	9.5
PBE0 TZ	55.9	19.5	16.0	15.1	60.7	22.3
PBE0 QZ	52.2	17.7	15.1	9.9	55.6	23.6
PBE TZ	55.2	29.4	17.2	56.1	59.4	40.9
PBE QZ	51.0	27.5	16.4	48.4	55.0	43.0
PW6B95 TZ	30.4	15.1	10.5	7.1	35.8	6.0
PW6B95 QZ	25.8	13.1	9.2	8.8	30.4	6.3
SCAN TZ	39.7	20.3	14.5	17.2	42.1	14.0
SCAN QZ	36.8	18.4	13.7	16.6	37.5	13.6

**Table 5:** Mean Absolute Errors and Mean Absolute Percentage Errors for Frequencies Compared to CCSD­(T) Calculations According to Species Containing Bonds of Type “Si–Si”, “O–H”, “Si–H”, “Si–C”, “Si–O” or “C–H”, Where the Number in Parentheses Is the Mean Absolute Percentage Error (MAPE)

	**MAE (MAPE) of Frequencies (cm** ^**–1**^ **(%))**					
**Method**	**Si–Si**	**O–H**	**Si–H**	**Si–C**	**Si–O**	**C–H**
B2GP-PLYP TZ	13.3 (1.7)	16.1 (1.0)	21.7 (2.1)	32.0 (10.7)	10.7 (3.8)	28.7 (2.0)
B2GP-PLYP QZ	14.9 (1.8)	17.3 (0.9)	24.9 (2.3)	33.3 (10.5)	10.9 (3.5)	28.9 (2.0)
B3LYP TZ	9.0 (2.1)	18.6 (1.3)	17.9 (2.2)	35.6 (12.3)	14.6 (4.0)	20.6 (1.9)
B3LYP QZ	7.4 (1.9)	16.2 (1.1)	14.4 (2.0)	33.6 (11.3)	12.8 (3.6)	20.8 (1.9)
M06 TZ	17.2 (2.7)	19.5 (1.3)	17.6 (2.2)	36.4 (13.8)	20.1 (6.0)	31.1 (2.9)
M06 QZ	18.4 (2.5)	20.4 (1.3)	19.7 (2.0)	38.2 (13.9)	21.2 (5.9)	28.4 (2.8)
M06-2X TZ	19.9 (2.7)	19.6 (1.2)	24.3 (2.3)	44.4 (12.7)	20.8 (5.0)	28.7 (2.6)
M06-2X QZ	21.2 (2.9)	19.4 (1.1)	23.9 (2.2)	44.6 (12.3)	22.7 (5.2)	28.2 (2.6)
M11 TZ	13.2 (2.8)	21.9 (2.0)	19.9 (2.4)	48.2 (14.8)	19.6 (6.2)	26.6 (2.7)
M11 QZ	20.1 (3.3)	38.3 (2.7)	36.4 (3.2)	51.4 (15.3)	28.8 (6.8)	28.1 (2.7)
PBE0 TZ	10.5 (1.6)	16.4 (1.1)	20.3 (2.1)	34.5 (11.5)	15.1 (4.9)	20.3 (1.9)
PBE0 QZ	9.6 (1.6)	14.9 (1.0)	16.9 (1.8)	34.5 (11.3)	14.3 (4.7)	20.3 (1.9)
PBE TZ	38.1 (5.4)	68.4 (4.1)	61.3 (5.1)	37.4 (9.3)	48.3 (7.8)	55.2 (3.3)
PBE QZ	35.0 (4.9)	66.4 (3.9)	56.6 (4.8)	36.1 (9.1)	45.7 (7.3)	55.8 (3.3)
PW6B95 TZ	10.1 (1.5)	13.1 (0.8)	13.9 (1.6)	34.6 (11.5)	12.9 (4.4)	22.0 (1.9)
PW6B95 QZ	11.5 (1.6)	13.7 (0.8)	14.1 (1.5)	35.5 (11.2)	13.1 (4.3)	22.1 (1.9)
SCAN TZ	10.6 (1.9)	16.0 (1.1)	20.6 (2.2)	25.9 (4.9)	14.4 (2.4)	17.8 (1.6)
SCAN QZ	9.0 (1.7)	14.2 (1.0)	16.7 (2.0)	26.7 (5.0)	12.9 (2.1)	17.8 (1.6)

As for the overall picture, M06-2X gives the lowest MAE in formation enthalpies for all bond types except for molecules with Si–C bonds or C–H bonds (see Table 4). In the former case PW6B95 has the smallest MAE at 8.8 kJ/mol followed by PBE0 (9.9 kJ/mol), B2GP-PLYP (13.4 kJ/mol), M06-2X (15.7 kJ/mol), SCAN (16.6 kJ/mol), and B3LYP (16.9 kJ/mol). PBE0 has the smallest ME (−0.1 kJ/mol) for Si–C where PW6B95, SCAN, and M06-2X also have ME rather close to zero at −2.7, −6.6, and −7.8 kJ/mol, respectively (Table S3). B3LYP has a ME for Si–C species of 16.9 kJ/mol indicating a consistent overestimate of the formation enthalpy. The largest differences in the MAE of the formation enthalpy are found for molecules containing Si–Si bonds. The M06-2X functional outperforms the other functionals with a MAE of 8.7 kJ/mol and is followed by M06, B2GP-PLYP, and PW6B95 with 24.3, 24.8, and 25.8 kJ/mol, respectively. B3LYP, which has reasonable overall MAE values, has in this case the highest MAE of 58.8 kJ/mol, which is even higher than for PBE (51.0 kJ/mol) and PBE0 (52.2 kJ/mol). Molecules with Si–O bonds exhibit a rather large spread in MAE values, where PBE0, PBE, B3LYP, and SCAN give rather high MAEs of 55.6, 55.0, 49.0, and 37.5 kJ/mol, respectively, compared to 10.8 kJ/mol for M06-2X and 12.2 kJ/mol for M06. The B3LYP functional has considerably lower MAE for molecules containing Si–H (17.5 kJ/mol), Si–C (16.9 kJ/mol), O–H (19.9 kJ/mol) and C–H (10.1 kJ/mol) bonds. The C–H bonds seem to be best described by B2GP-PLYP (MAE: 5.2 kJ/mol), together with PW6B95 (6.3 kJ/mol), M06-2X (6.7 kJ/mol), M06 (8.5 kJ/mol), M11 (9.5 kJ/mol), and B3LYP (10.1 kJ/mol). For the O–H bonds, the Minnesota functionals together with B2GP-PLYP are remarkably better than the other functionals. M06-2X, M11, B2GP-PLYP, and M06 show small MAE values, i.e., 4.4, 5.0, 6.9, and 9.3 kJ/mol, respectively, and are followed by PW6B95 (13.1 kJ/mol), PBE0 (17.7 kJ/mol) and SCAN (18.4 kJ/mol). M06-2X has the smallest MEs for all bonds, apart from Si–C as discussed above, Si–H, where it is close to SCAN, and Si–O, where it is close to M06 (Table S3). The second smallest ME for Si–Si is found for M06, for C–H it is B2GP-PLYP, and for O–H it is M11. The PW6B95 and SCAN functionals give small MEs for species with Si–C, Si–H and C–H bonds.

Regarding vibrational frequencies (Table 5), SCAN shows the smallest MAEs for Si–C (26.7 cm^–1^) and C–H bonds (17.8 cm^–1^), B3LYP has the smallest MAEs for Si–Si (7.4 cm^–1^) and Si–H (14.4 cm^–1^), B2GP-PLYP performs best for Si–O (10.9 cm^–1^), and PW6B95 for O–H (13.7 cm^–1^). PBE0 generally provides among the smallest MAEs and has the smallest ME (see Table S4) for several bond types (Si–Si, Si–H, and Si–O). PBE shows the poorest performance, with MAEs of 35.0 cm^–1^ and higher. The Minnesota functionals also perform relatively poorly with MAEs ranging from 18.4 cm^–1^ (M06 for Si–Si) and 19.4 cm^–1^ (M06-2X for O–H) to 44.6 and 51.4 cm^–1^ (M06-2X and M11 for Si–C). The most challenging bond type seems to be Si–C where SCAN performs somewhat better (MAE: 26.7 kJ/mol) than B2GP-PLYP, B3LYP, PBE0, and PW6B95 with MAEs of 33.3, 33.6, 34.5, and 35.5 cm^–1^, respectively. SCAN shows a more balanced description of Si–C frequencies (Table S4) with an ME of 6.4 cm^–1^ compared to B3LYP (14.4 cm^–1^), PBE0 (25.9 cm^–1^), PW6B95 (28.9 cm^–1^), and B2GP-PLYP (29.1 cm^–1^). Si–C is the only bond type for which PBE does not show the poorest performance for frequencies among the studied functionals (MAE: 36.1 cm^–1^; ME: −23.0 cm^–1^). The results for zero-point energies (Table S5) show the same general trends for the different bond types, with SCAN, B3LYP, PBE0, and PW6B95 providing the best performance. The MAPEs (Table 5) also reveal some more details of the different bond-types. SCAN has the smallest MAPE for Si–C (5.0%), Si–O (2.1%), and C–H (1.6%), PW6B95 shows best performance for O–H (0.8%) and Si–H (1.5%), and PBE0 and PW6B95 both have MAPEs of 1.6% for Si–Si. For Si–C, PBE has actually the second lowest MAPE (9.1%) followed by B2GP-PLYP (10.5%).

### Reaction Energies

To evaluate how the density functionals behave for calculating the relative stabilities of species within the same reaction system, all theoretically possible reactions (369 in total), only considering reaction stoichiometry, have been defined, connecting the species studied here (see Tables S6 and S7 for the complete list of reactions). For isomeric species only the lowest energy isomer was included as reactant in the full reaction scheme, and the other higher-energy isomers were only included in unimolecular isomerization reactions. Since C_2_ was excluded from the original benchmark data set, all reactions including C_2_ were removed. In addition, in reaction systems where a set of reactants can lead to several different products, only the product giving the smallest reaction energy was included in the benchmark reaction data set. Thereby all the reactions and reaction energies included as benchmarks are linearly independent; i.e., two reactions in the data set cannot be combined to yield a third reaction already in the data set. The remaining 200 reactions in the benchmark data set are presented in Table S6 and the discarded 169 reactions are shown in Table S7. Most of the reactions are bimolecular reactions with two products and, in addition, a few unimolecular isomerization reactions as well as some clustering reactions, i.e., two reactants and one product, for the Si–O species. For each reaction, the reaction energy (including zero-point energy) at the CCSD­(T) level was defined as the benchmark. All reactions are defined such that they are exothermic (at 0 K); i.e., the reaction energies are all negative.

To gain further insight into the capabilities of the density functionals for different chemical compositions, the reactions have been organized into subclasses depending on the composition of reactants and products. The different types of reactions are bimolecular and unimolecular Si–O, Si–C, Si–H, Si–O–H, Si–C–H, and Si–C–O reactions and Si–O clustering reactions. Results are shown in Table 6 and Table S8, and the performance for the different reactions are discussed below.

**Table 6:** Mean Absolute Errors and Mean Errors (in kJ/mol) of Reaction Energies (Including Zero-Point Energy) for the Si–C–O–H System Overall and Divided in Composition of Reaction System for All of the Reactions Listed in Table S6

	**Mean Absolute Error (MAE)**							
**Method**	**Overall**	**Si–C**	**Si–C–H**	**Si–C–O**	**Si–H**	**Si–O**	**Si–O–H**	Si–O Cluster
B2GP-PLYP	7.4	8.2	7.2	7.8	8.4	7.3	7.1	7.9
B3LYP	13.8	14.9	13.1	18.8	16.8	17.3	13.1	15.6
M06	12.3	13.1	13.3	13.8	12.1	12.6	9.8	11.7
M06-2X	15.0	15.9	14.4	17.7	14.4	17.7	13.7	17.5
M11	14.7	18.4	13.7	18.7	15.1	16.9	12.8	13.8
PBE0	17.8	16.4	17.8	22.4	20.3	21.0	16.9	19.2
PBE	24.0	27.4	22.0	28.8	23.9	25.8	22.4	26.9
PW6B95	9.9	9.9	9.2	14.2	11.8	13.3	9.5	12.5
SCAN	22.2	22.6	20.9	22.8	20.0	21.0	18.6	17.8

	**Mean Error (ME)**							
**Method**	**Overall**	**Si–C**	**Si–C–H**	**Si–C–O**	**Si–H**	**Si–O**	**Si–O–H**	Si–O Cluster
B2GP-PLYP	1.5	0.8	1.6	3.3	2.0	2.7	1.8	5.7
B3LYP	6.9	6.3	6.6	10.6	7.4	8.2	6.6	10.1
M06	3.8	0.3	3.2	3.4	4.1	3.0	2.3	0.8
M06-2X	–1.1	–3.8	–0.3	1.2	1.1	–0.1	–2.3	–2.5
M11	0.2	–4.2	0.6	2.9	1.8	1.5	–0.2	–1.4
PBE0	8.4	3.2	7.2	12.6	9.5	10.6	7.8	8.8
PBE	7.9	6.5	5.4	12.3	6.9	9.5	6.1	11.8
PW6B95	4.1	0.7	3.8	6.8	5.2	5.5	3.7	5.4
SCAN	11.0	10.4	8.3	13.3	8.6	9.5	8.0	7.1

Overall, the best performing functionals for these reaction energies are B2GP-PLYP (MAE: 7.4 kJ/mol), PW6B95 (9.9 kJ/mol), followed by the Minnesota functionals (M06: 12.3 kJ/mol; M11: 14.7 kJ/mol; M06-2X: 15.0 kJ/mol), and B3LYP (13.8 kJ/mol). M11, M06-2X, and B2GP-PLYP have relatively small MEs at 0.2, −1.1, and 1.5 kJ/mol, respectively, and they do not show any systematic under- or overestimation of the reaction energies, as the other functionals do. There is a tendency among the other functionals to somewhat overestimate the reaction energies but not by more than, on average, 11.0 kJ/mol (as for SCAN). PBE shows the largest MAE followed by SCAN. Since these are the only nonhybrid functionals, that result should not be overly surprising considering the general success of hybrid functionals for the calculation of reaction energies.

### Si–O Reactions, Si–O Clustering, and Si–O–H Reactions

In the recent studies by Andersson et al.^37,38^ it was concluded that the M06 functional gave excellent agreement with CCSD­(T) calculations for the potential energy surfaces (PES) of small silicon oxide molecules reacting with OH and H_2_O (including intermediate energies, barrier heights, and reaction energies). For the more general class of reaction energies for Si–O and Si–O–H reactions it is seen that M06 has the second smallest MAE (12.6 kJ/mol) for Si–O reactions after B2GP-PLYP (7.3 kJ/mol) and third smallest MAE (9.8 kJ/mol), after B2GP-PLYP (7.1 kJ/mol) and PW6B95 (9.5 kJ/mol) for Si–O–H. Since B2GP-PLYP and PW6B95 were not previously considered for these reactions, the results are in line with the previous studies on reactions of Si–O–H species. Other reasonable MAEs are provided by M06-2X (Si–O: 17.7 kJ/mol; Si–O–H: 13.7 kJ/mol), M11 (16.9 and 12.8 kJ/mol), and B3LYP (17.3 and 13.1 kJ/mol). M06-2X, M11, M06, B2GP-PLYP have MEs around zero, but in general all six of these functionals should be a reasonable choice for the study of these types of reactions. In the case of Si–O clustering reactions, B2GP-PLYP, M06, and PW6B95 again have the smallest MAEs at 7.9, 11.7, and 12.5 kJ/mol, respectively. Taking the enthalpies of formation and vibrational frequencies into account in addition, it seems that PW6B95 would be a preferred choice for reaction thermochemistry, since its errors in Δ*H*_f,0_^0^ for molecules containing Si–O, Si–H, Si–Si, and O–H bonds are overall the third smallest after M06-2X and B2GP-PLYP, but scores highest for these bonds when it comes to all three measures of vibrational frequencies, while M06-2X is among the three poorest of the studied functionals and B2GP-PLYP shows average performance for frequencies.

### Si–C, Si–C–H, Si–C–O, and Si–H Reactions

For these types of reactions, the picture is similar to that of the previous class of reactions. B2GP-PLYP has the lowest MAE for all reactions (Si–C: 8.2 kJ/mol, Si–C–H: 7.2 kJ/mol, Si–C–O: 7.8 kJ/mol, Si–H: 8.4 kJ/mol), with PW6B95 having the second lowest MAE for Si–C (9.9 kJ/mol), Si–C–H (9.2 kJ/mol), and Si–H (11.8 kJ/mol), and M06 having the second lowest MAE for Si–C–O (13.8 kJ/mol). For the Si–C–H, Si–C–O, and Si–H reactions, M06-2X and M11 have the smallest MEs (M06-2X: −0.3, 1.2, 1.1 kJ/mol; M11: 0.6, 2.9, 1.8 kJ/mol), but for the Si–C reactions, M06 has the smallest ME (0.3 kJ/mol) and PW6B95 has the second smallest at 0.7 kJ/mol. Again taking Δ*H*_f,0_^0^ and the frequencies into account, it is seen that B3LYP is among the best for Si–Si, Si–C, and Si–H frequencies, but performs especially poorly for the Si–C Δ*H*_f,0_^0^, while M06-2X performs best for the formation enthalpies. Since the enthalpies of formation of Si–C species were well described by PBE0, one would expect that reaction energies for the reactions involving these species would also be well treated by PBE0. However, also in the cases of Si–C, Si–C–H, and Si–C–O reactions, PBE0 is on the lower half of the scoreboard together with PBE and SCAN. Also for these reaction systems, PW6B95 seems to provide the best-balanced description of the properties evaluated here, as it scores highly in all categories.

## Conclusions

This study compared the performance of different density functionals (B2GP-PLYP, B3LYP, M06, M06-2X, M11, PBE0, PBE, PW6B95, and SCAN) for the calculation of enthalpies of formation, vibrational frequencies, and reaction energies for molecular species with general compositions Si–H, Si–O, Si–O–H, Si–C, Si–C–H, and Si–C–O with coupled cluster [CCSD­(T)] results as reference values.

For this set of species, B2GP-PLYP, PW6B95, and the Minnesota functionals (M06-2X, M06 and M11) perform better for enthalpies of formation than SCAN, B3LYP and PBE0 and significantly better than PBE. Vibrational frequencies are best described by SCAN followed by PW6B95, B3LYP, and PBE0 with PBE being relatively poor. Some variations are noted when the results are sorted into different bond types (Si–Si, O–H. Si–H, Si–C, Si–O, and C–H). Si–C bonds are the most challenging type for frequencies, and Si–Si and Si–O bonds pose a challenge for calculating enthalpies of formation. PW6B95 and PBE0 perform best for enthalpies of formation of Si–C species. For all other considered bond types, M06-2X shows the best performance for enthalpy of formation, except for molecules with C–H bonds, which are best described by B2GP-PLYP. SCAN performs best for vibrational frequencies and zero-point energies overall, but PW6B95, B3LYP and PBE0 all show similar performances for Si–Si, Si–H, and C–H bonds, B2GP-PLYP for Si–C and Si–O bonds, PW6B95 for O–H bonds, and B3LYP for Si–C bonds. The worst performance for enthalpy of formation for each bond type is found for PBE except for Si–Si bonds (B3LYP), Si–H bonds (M06), and Si–O bonds (PBE0). The corresponding poorest performer for frequencies is PBE for all bond types except Si–C where M11 has the largest errors for frequencies and zero-point energies.

Finally, the performance for possible reaction energies within the Si–O–C–H systems were evaluated. B2GP-PLYP, PW6B95, and M06 gave the best results and had somewhat lower MAEs than M06-2X and M11, which in turn showed a more even distribution of under- and overestimation of reaction energies (mean error of about 0 kJ/mol for both). PBE, PBE0 and SCAN showed generally poorer performance for reaction energies, whereas B3LYP results were in between those two groups.

General recommendations for the use of the studied density functionals on Si–O–C–H systems would be to use M06-2X for enthalpies of formation, SCAN for vibrational frequencies and zero-point energies, and B2GP-PLYP for reaction energies. For the enthalpies of formation for pure Si–C-containing molecules, PBE0 would be a good choice, but for their reaction energies, B2GP-PLYP, PW6B95, and M06 perform better. PW6B95 was the density functional that most consistently performed well overall for enthalpies of formation, vibrational frequencies, and reaction energies and is therefore recommended as a suitable starting point for DFT calculations on Si–O–C–H systems.

## Supplementary Material



## Notes

Figures of MAEs, MEs, and individual errors for all DFT calculations; table of calculated [CCSD­(T)] and experimental vibrational frequencies; tables of MAEs and MEs for all DFT calculations for enthalpy of formation, vibrational frequencies, and zero-point frequencies (overall and divided into bond types); table of all calculated reaction energies [CCSD­(T)]; table of MAEs and MEs for reaction energies for all DFT calculations; XYZ coordinates for all included molecular species as calculated by CCSD­(T) (PDF)
