# Supplementary material for: Evaluation of Density Functionals for Si–O–C–H Molecule Thermochemistry
Source: J Phys Chem A. 2025 Oct 22;129(44):10251–62. doi: 10.1021/acs.jpca.5c04844 (PMC12598855; doi:10.1021/acs.jpca.5c04844)
Supplement: Supplementary file 1 [file jp5c04844_si_001.pdf]

Supporting information

Evaluation of Density Functionals for Si-O-C-H Molecule Thermochemistry

Ingeborg-Helene Svenum[a], Francesca Lønstad Bleken[b], Stefan Andersson[a]

[a] SINTEF Industry, P. O. Box 4760 Torgarden, Trondheim 7465, Norway

[b] SINTEF Industry, P. O. Box 124 Blindern, Oslo 0314, Norway

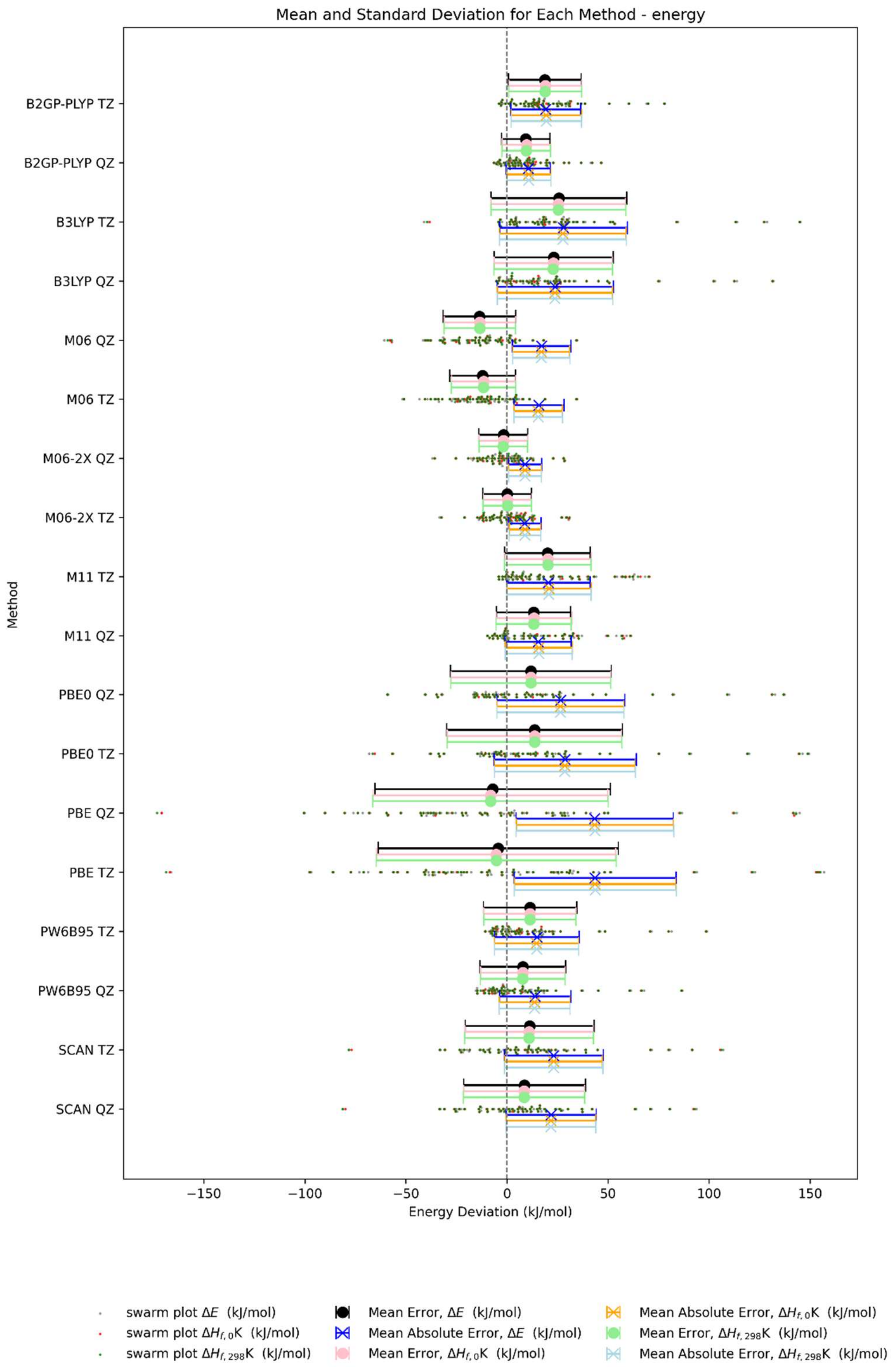

Figure S1. Mean absolute error and mean error for the calculated formation energies for selected functionals. Errors are calculated with respect to the coupled cluster results, and standard deviations are included as error bars. Individual errors are also indicated in the plot.

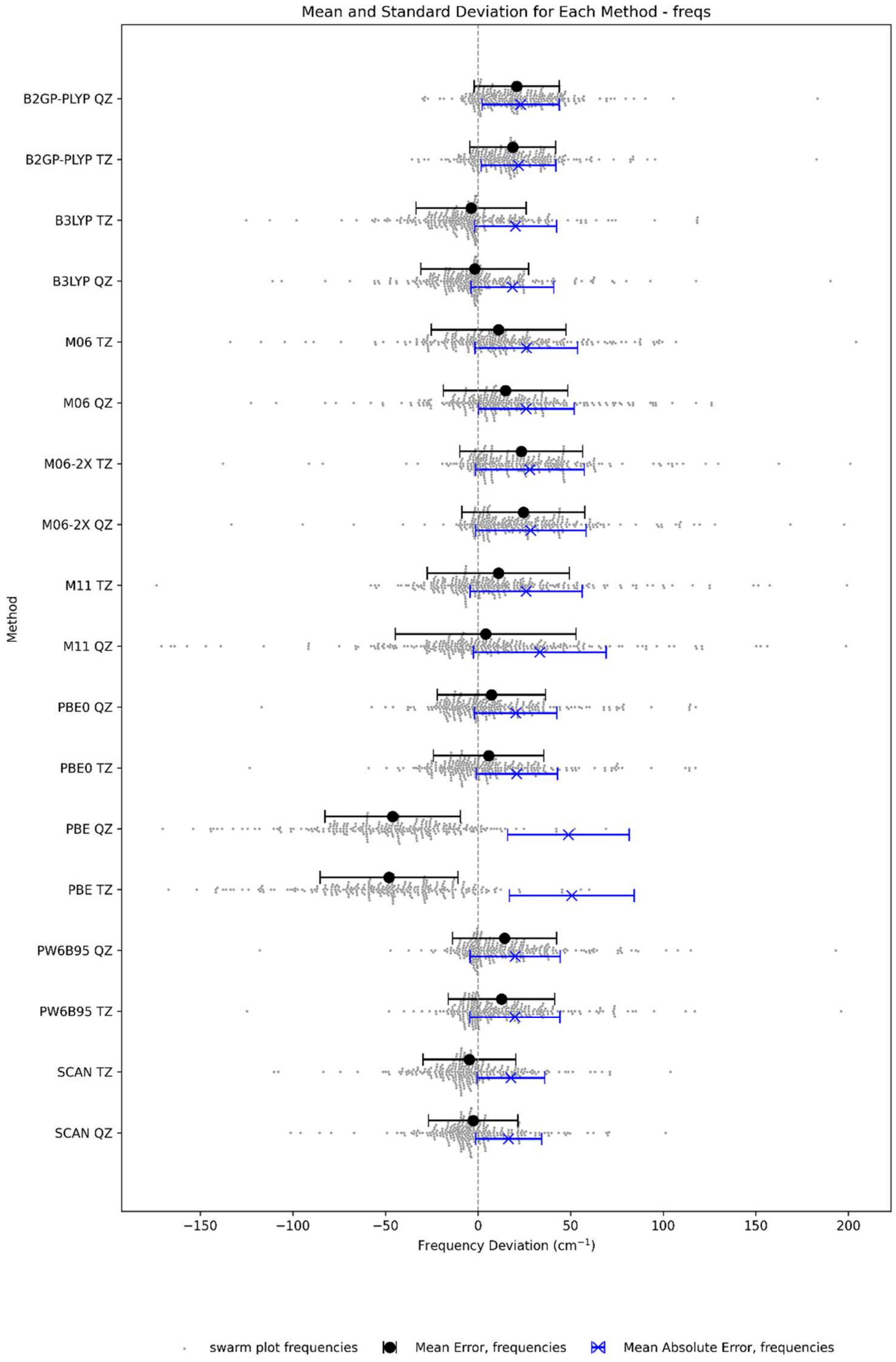

Figure S2. Mean absolute error and mean error for the calculated frequencies in cm^{-1} for selected functionals. Errors are calculated with respect to the coupled cluster results, and standard deviations are included as error bars. Individual errors are also indicated in the plot.

Table S1. Percentage of total atomization energy (TAE) accounted for by triples [(T)] excitations. (M = spin multiplicity).

Species	M	%TAE[(T)]
O_2	3	7.0
CO	1	3.1
CO_2	1	3.6
C_2	1	13.4
C_2	3	6.4
C_3	1	5.6
SiO	1	4.4
SiO_2	1	4.9
Si_2	3	5.8
Si_3	1	8.6
Si_2O	1	4.5
Si_2O_2	1	3.1
SiC	3	6.0
SiC_2	1	4.4
CSi_2	1	5.2
Si_2C_2	1	4.2
SiCO	3	3.9
CSiO	3	5.9
OH	2	1.6
H_2O	1	1.5
CH	2	1.0
CH_2	3	0.5
CH_3	2	0.6
CH_4	1	0.7
SiH	2	0.5
SiH_2	1	0.6
SiH_3	2	0.3
SiH_4	1	0.3
H_2SiC	1	2.9
H_2CSi	1	2.1
HCSiH	1	4.1
HOSi	2	2.4
HSiO	2	3.8
H_2SiO	1	2.4
cis-HSiOH	1	1.8
trans-HSiOH	1	1.9
H_2CO	1	2.1
HCO	2	3.0
H_3SiOH	1	1.6
Si_2O_3	1	3.3
Si_2O_4	1	3.5
Si_3O_3	1	2.9
Si_2C_2_linear	1	5.2
SiC_3_linear	1	4.9
C_2H_2	1	2.1
C_2H_4	1	1.3
C_2H_6	1	0.9
SiH_3SiH_3	1	0.6
SiH_3SiH	1	0.9

Table S2. Harmonic vibrational frequencies (in cm^{-1}) calculated with CCSD(T)/aug-cc-pV(Q+d)Z and experimental harmonic and fundamental frequencies from literature. (M = spin multiplicity).

Species	M	Frequencies CCSD(T)	Experimental frequencies harmonic	Experimental frequencies fundamental	Reference
O_2	3	1595.7	1580.161	1556.4	[1]
CO	1	2160.1	2169.75589	2143.2	[1]
CO_2	1	2388.3	2396.4	2349	[1]
		1349.3	1351.2	1333	
		669.5	672.2	667	
		669.5			
C_2	1	1856.6	1855.0663	1827.5	[1]
C_2	3	1639.6			
C_3	1	2117.9		2040	[1]
		1215.9		1224.5	[1]
		73.6		63.4	[1]
		73.6			
SiO	1	1233.4	1241.54388	1229.6	[1]
SiO_2	1	1432.1		1416.4	[1]
		986.9			
		289.3		272.5	[1]
		289.3			
Si_2	3	514.3	510.98	506.9	[1]
Si_3	1	557.8		550.6	[2]
		531.9		525.5	[2]
		185.3		178±11	[3]
Si_2O	1	829.8			
		634.8			
		441.8			
Si_2O_2	1	855.9			
		824.3		803.2	[4]
		780.5		768.2	[4]
		577.9			
		558.2			
		237.5			
SiC	3	985.0	964.6	953.4	[1]
SiC_2	1	1764.7	1756.8	1746	[1]
		808.8	844	840.6	[1]
		180.1		196.4	[1]
CSi_2	1	1213.6		1188.4	[5]
		847.8		839.5	[5]
		149.0		166.4	[5]
Si_2C_2	1	1074.2			
		998.9			
		976.7		982.9	[6]
		519.7			

		383.2		382.2	[6]
		198.5			
SiCO	3	1950.7		1898.1	[7]
		567.5			
		344.0			
		344.0			
CSiO	3	1246.5			
		667.2			
		51.9			
		51.9			
CH	2	2853.8	2860.7508	2733.1	[1]
CH_2	3	3370.5		3190	[1]
		3142.3		2805.9	[1]
		1098.0		963.1	[1]
CH_3	2	3302.0		3160.8	[1]
		3302.0			
		3118.9		3004.4	[1]
		1420.9		1396	[1]
		1420.9			
		509.5		606.5	[1]
CH_4	1	3153.7	3156.8	3019	[1],[8],[9]
		3153.7			
		3153.7			
		3032.0	3025.5	2917	[1],[8],[9]
		1569.7	1582.7	1534	[1],[8],[9]
		1569.7			
		1344.4	1367.4	1306	[1],[8],[9]
		1344.4			
		1344.4			
SiH	2	2046.0	2042.5229	1970.8	[1]
SiH_2	1	2074.2		1995.9	[1]
		2072.3		1992.8	[1]
		1020.4		999	[1]
SiH_3	2	2261.6		2185.2	[1]
		2261.6			
		2229.4		2136	[1]
		940.7		922	[1]
		940.7			
		770.0		727.9	[1]
SiH_4	1	2264.2		2191	[1]
		2264.1		2187	[1]
		2264.1			
		2260.9			
		1026.8		975	[1]
		955.4			
		931.9			

		927.0		914	[1]
		899.1			
H_2SiC	1	2287.5			
		2250.9			
		936.2			
		804.0			
		335.5			
		300.3			
H_2CSi	1	3168.3			
		3085.5		2947±5	[10]
		1344.3		1273±3	[10]
		934.5		933±3	[10]
		693.0		687±5	[10]
		311.8		263±5	[10]
HCSiH	1	3276.8			
		2253.4			
		1072.5			
		722.0			
		602.4			
		450.3			
HOSi	2	3844.6			
		877.0			
		771.4			
HSiO	2	1959.0			
		1178.2			
		652.0			
H_2SiO	1	2266.8			
		2252.0			
		1215.4		1202	[11]
		1008.4			
		702.7		697	[11]
		684.7			
cis-HSiOH	1	3870.6		3661	[11]
		1967.6		1846/1882	[11]
		970.0		938	[11]
		864.6		847	[11]
		749.2		723	[11]
		628.5		596	[11]
trans-HSiOH	1	3867.9		3661	[11]
		2044.0		1846/1882	[11]
		966.8		938	[11]
		861.3		847	[11]
		806.4		723	[11]
		666.4		596	[11]
H_2CO	1	3006.4	3012.0	2843	[1],[8],[12]
		2935.2	2937.4	2782	[1],[8],[12]

		1774.9	1777.8	1746	[1],[8],[12]
		1533.3	1544.0	1500	[1],[8],[12]
		1268.4	1269.4	1249	[1],[8],[12]
		1185.1	1188.3	1167	[1],[8],[12]
HCO	2	2705.2		2434.5	[1]
		1893.7		1868.2	[1]
		1115.4		1080.8	[1]
H_2	1	4399.6	4401.213	4161.2	[1]
OH	2	3739.2	3737.761	3569.6	[1]
H_2O	1	3940.5	3942.5	3756	[1]
		3830.8	3832.2	3657	[1]
		1650.0	1648.5	1595	[1]
H_3SiOH	1	3901.1			
		2273.6			
		2232.1			
		2225.1			
		1000.0			
		984.6			
		964.6			
		908.1		859	[11]
		847.6			
		723.1			
		687.0			
		201.4			
Si_2O_3	1	1322.4			
		863.9			
		863.1			
		805.9			
		646.0			
		522.4			
		412.1			
		278.1			
		168.1			
Si_2O_4	1	1337.1			
		1299.8		1293.3	[13]
		893.3		889.2	[13]
		859.5			
		792.4		786.4	[13]
		709.0			
		482.0			
		450.6		442	[13]
		309.7			
		296.6		285	[13]
		235.0			
		122.3			
Si_3O_3	1	1000.9			

		998.6		972.6	[14]
		779.8			
		635.8		631.5	[14]
		634.8			
		604.8			
		540.2			
		317.7		312.0	[14]
		316.2			
		216.9			
		112.8			
		112.7			
Si_2C_2_linear	1	1748.0			
		892.7			
		463.7			
		406.5			
		260.8			
		137.0			
		93.2			
SiC_3_linear	1	1918.7		1980±20	[15]
		1296.9		1320±20	[15]
		602.2			
		280.9			
		280.9			
		114.0		130	[15]
		114.0			
C_2H_2	1	3499.0	3495.1	3374	[1],[8],[16]
		3407.0	3415.2	3289	[1],[8],[16]
		2004.2	2007.6	1974	[1],[8],[16]
		743.1	746.7	730	[1],[8],[16]
		742.9			
		603.2	624.0	612	[1],[8],[16]
		603.1			
C_2H_4	1	3246.7	3251	3105	[1],[8],[17]
		3220.5	3226	3086	[1],[8],[17]
		3154.2	3163	3026.4	[1],[8],[17]
		3138.4	3149	2988.7	[1],[8],[17]
		1669.0	1676	1622.9	[1],[8],[17]
		1475.8	1482	1443.5	[1],[8],[17]
		1367.1	1366	1342.2	[1],[8],[17]
		1246.2	1241	1217	[1],[8],[17]
		1046.9	1046	1023	[1],[8],[17]
		964.4	967	949.3	[1],[8],[17]
		948.9	942	939.6	[1],[8],[17]
		823.9	823	826	[1],[8],[17]
C_2H_6	1	3119.9		2985	[1]
		3119.9			

		3096.8	2969	[1]
		3096.8		
		3036.6	2954	[1]
		3036.3	2896	[1]
		1512.2	1469	[1]
		1512.2		
		1510.6	1468	[1]
		1510.6		
		1426.7	1388	[1]
		1408.5	1379	[1]
		1224.8	1190	[1]
		1224.8		
		1013.5	995	[1]
		820.6	822	[1]
		820.6		
		306.8	289	[1]
SiH_3SiH_3	1	2233.7	2178.6	[1]
		2233.7		
		2225.5	2155	[1]
		2224.8	2154.3	[1]
		2224.8		
		2216.8	2152	[1]
		959.8	939.6	[1]
		959.8		
		946.0	929.3	[1]
		946.0		
		928.2	909	[1]
		854.1	843.5	[1]
		634.4	625.2	[1]
		634.4		
		436.9	434.2	[1]
		369.6	379.3	[1]
		369.6		
		138.4	131	[1]
SiH_3SiH	1	2220.1		
		2196.6		
		2188.5		
		2041.6		
		958.2		
		934.7		
		871.5		
		709.4		
		428.2		
		382.3		
		374.7		
		97.1		

[1] NIST Computational Chemistry Comparison and Benchmark Database, NIST Standard Reference Database Number 101 Release 22, May 2022, Editor: Russell D. Johnson III, http://cccbdb.nist.gov/ DOI:10.18434/T47C7Z
[2] Li, S.; Van Zee, R. J.; Weltner, W., Jr.; Raghavachari, K. Si_3-Si_7. Experimental and theoretical infrared spectra. *Chem. Phys. Lett.* **1995**, *243*, 275-280.
[3] McCarthy, M. C.; Thaddeus, P. Rotational Spectrum and Structure of Si_3. *Phys. Rev. Lett.* **2003**, *90*, 213003.
[4] Andrews, L.; McCluskey, M. Bending modes of SiO_2 and GeO_2 in solid argon. *J. Molec. Spectrosc.* **1992**, *154*, 223–225.
[5] Presilla-Márquez, J. D.; Graham, W. M. L. Fourier transform vibrational spectroscopy of Si_2C in solid Ar. *J. Chem. Phys.* **1991**, *95*, 5612 – 5617.
[6] Presilla-Márquez, J. D.; Gay, S. C.; Rittby, C. M. L.; Graham, W. M. L. Vibrational spectra of tetra-atomic silicon–carbon clusters. II. Si2C2 in Ar at 10 K. *J. Chem. Phys.* **1995**, *102*, 6354 – 6361.
[7] Reactions of silicon atoms and small clusters with CO: Experimental and theoretical characterization of Si_nCO (n=1 – 5), $Si_2(CO)_2$, c-SiO_2 (μ-O)(μ-CSi) and c-Si_2 (μ-O)(μ-CCO) in solid argon. *J. Chem. Phys.* **2004**, *121*, 10474 – 10482.
[8] Martin, J. M. L.; El-Yazal, J; François, J.-P. Basis set convergence and performance of density functional theory including exact exchange contributions for geometries and harmonic frequencies. *Mol. Phys.* **1995**, *86*, 1437–1450.
[9] Gray, D. L.; Robiette, A. G. The anharmonic force field and equilibrium structure of methane. *Mol. Phys.* **1979**, *37*, 1901–1920.
[10] Smith, T. C.; Li, H.; Clouthier, D. J. The ground state of silylidene (H_2C=Si), the silicon analog of vinylidene, from stimulated emission pumping and wavelength-resolved fluorescence spectroscopy. *J. Chem. Phys.* **2001**, *114*, 9012 – 9019.
[11] Withnall, R.; Andrews, L. Matrix reactions of silane and oxygen atoms. Infrared spectroscopic evidence for the silanol, silanone, and silanoic and silicic acid molecules. *J. Phys. Chem.* **1985**, *89*, 3261 – 3268.
[12] Harding, L. B.; Ermler, W. C. Polyatomic, Anharmonic, Vibrational-Rotational Analysis. Application to Accurate Ab Initio Results for Formaldehyde. *J. Comput. Chem.* **1985**, *6*, 13 – 27.
[13] Mehner, T.; Gocke, H.J.; Schunck, S.; Schnockel, H. Dimeres SiO_2 Matrix-IR-Untersuchungen und ab initio SCF-Rechnungen, *Z. Anorg. Allg. Chem.* **1990**, *580*, 121 – 130.
[14] Anderson, J. S.; Ogden, J. S. Matrix Isolation Studies of Group-IV Oxides. I. Infrared Spectra and Structures of SiO, Si_2O_2, and Si_3O_3. *J. Chem. Phys.* **1969**, *51*, 4189 – 4196.
[15] Davico, G.E.; Schwartz, R.L.; Lineberger, W.C. Photoelectron spectroscopy of C_3Si and C_4Si_2 anions. *J. Chem. Phys.* **2001**, *115*, 1789 – 1794.
[16] Strey, G.; Mills, I. M. Anharmonic force field of acetylene. *J. Molec. Spectrosc.* **1976**, *59*, 103–115.
[17] Martin, J. M. L.; Lee, T. J.; Taylor, P. R.; François, J.-P. The anharmonic force field of ethylene, C_2H_4, by means of accurate ab initio calculations. *J. Chem. Phys.* **1995**, *103*, 2589 – 2602.

Table S1. MAE and ME for $\Delta H_{f,0}$ (in kJ/mol) for all species (overall and divided into bond types) relative to coupled-cluster results. Number in parenthesis is the standard deviation.

	Overall		Si-Si		O-H		Si-H		Si-C		Si-O		C-H	
**Method**	MAE	ME	MAE	ME	MAE	ME	MAE	ME	MAE	ME	MAE	ME	MAE	ME
**B2GP-PLYP TZ**	19.5 (17.4)	19.0 (18.0)	39.8 (19.0)	39.8 (19.0)	12.5 (6.2)	12.5 (6.2)	14.2 (9.8)	14.0 (10.0)	25.8 (7.7)	25.8 (7.7)	31.2 (23.5)	31.2 (23.5)	9.8 (8.8)	8.9 (9.8)
**B2GP-PLYP QZ**	10.9 (11.0)	9.7 (12.0)	24.8 (12.1)	24.8 (12.1)	6.9 (3.0)	6.9 (3.0)	7.9 (6.5)	6.5 (8.0)	13.4 (5.9)	13.4 (5.9)	17.8 (15.1)	17.8 (15.1)	5.2 (5.6)	2.2 (7.4)
**B3LYP TZ**	27.7 (31.4)	25.5 (33.2)	64.1 (45.4)	64.1 (45.4)	22.2 (11.3)	22.2 (11.3)	20.2 (12.7)	19.7 (13.6)	24.0 (14.6)	17.0 (23.0)	54.8 (43.9)	54.8 (43.9)	11.8 (9.6)	9.8 (11.8)
**B3LYP QZ**	23.8 (28.6)	22.9 (29.3)	58.5 (40.9)	58.5 (40.9)	19.9 (9.6)	19.9 (9.6)	17.5 (11.4)	16.8 (12.5)	16.9 (13.6)	16.9 (13.6)	49.0 (39.7)	49.0 (39.7)	10.1 (8.2)	7.6 (10.8)
**M06 TZ**	15.4 (12.0)	-11.5 (15.9)	23.3 (13.9)	-8.9 (26.9)	9.3 (7.7)	-7.9 (9.3)	20.3 (10.9)	-19.1 (13.0)	22.7 (11.9)	-21.3 (14.4)	12.8 (8.5)	-1.6 (15.7)	7.6 (4.1)	-5.9 (6.5)
**M06 QZ**	17.0 (14.1)	-13.4 (17.6)	24.3 (14.2)	-10.8 (27.3)	9.3 (6.5)	-6.8 (9.5)	19.9 (11.6)	-19.4 (12.6)	28.5 (16.7)	-27.8 (17.9)	12.2 (9.0)	-1.9 (15.4)	8.5 (4.6)	-7.8 (5.8)
**M06-2X TZ**	9.0 (7.8)	0.3 (12.0)	10.6 (8.8)	1.4 (14.3)	3.9 (1.9)	-0.5 (4.7)	8.3 (7.8)	3.3 (11.1)	14.6 (9.6)	-5.1 (17.3)	10.6 (8.4)	4.3 (13.1)	6.9 (8.7)	3.0 (10.8)
**M06-2X QZ**	8.9 (8.1)	-1.7 (12.0)	8.7 (7.1)	0.0 (11.7)	4.4 (2.5)	-0.6 (5.4)	7.7 (7.2)	2.7 (10.5)	15.7 (10.1)	-7.8 (17.5)	10.8 (8.1)	2.5 (13.6)	6.7 (8.2)	-0.8 (10.8)
**M11 TZ**	20.8 (20.9)	20.2 (21.4)	38.8 (17.9)	38.8 (17.9)	4.6 (2.9)	2.4 (5.1)	14.4 (16.1)	14.0 (16.5)	34.6 (22.1)	34.6 (22.1)	28.2 (20.5)	28.2 (20.5)	12.1 (16.0)	11.4 (16.6)
**M11 QZ**	15.8 (16.6)	13.3 (18.8)	27.3 (16.1)	27.3 (16.1)	5.0 (3.8)	-5.0 (3.8)	10.2 (14.1)	6.5 (16.3)	28.8 (19.2)	27.6 (21.1)	18.4 (11.9)	15.9 (15.2)	9.5 (14.1)	8.0 (15.1)
**PBE0 TZ**	28.7 (34.8)	13.6 (43.1)	55.9 (56.4)	53.3 (59.2)	19.5 (9.5)	19.5 (9.5)	16.0 (11.5)	11.3 (16.5)	15.1 (17.8)	-1.5 (23.7)	60.7 (47.0)	60.7 (47.0)	22.3 (17.5)	-18.8 (21.6)
**PBE0 QZ**	26.5 (31.4)	11.9 (39.5)	52.2 (50.9)	48.5 (54.8)	17.7 (8.2)	17.7 (8.2)	15.1 (10.0)	9.1 (16.0)	9.9 (5.6)	-0.1 (11.8)	55.6 (43.2)	55.6 (43.2)	23.6 (17.6)	-20.7 (21.3)
**PBE TZ**	43.6 (39.9)	-5.4 (59.2)	55.2 (58.4)	43.5 (68.8)	29.4 (7.5)	29.4 (7.5)	17.2 (14.6)	6.4 (22.1)	56.1 (40.8)	-56.1 (40.8)	59.4 (49.5)	54.6 (55.0)	40.9 (20.1)	-40.9 (20.1)
**PBE QZ**	43.5 (38.8)	-8.1 (58.0)	51.0 (54.0)	39.5 (64.1)	27.5 (6.7)	27.5 (6.7)	16.4 (14.3)	4.3 (21.8)	48.4 (19.3)	-48.4 (19.3)	55.0 (45.7)	49.9 (51.7)	43.0 (20.6)	-43.0 (20.6)
**PW6B95 TZ**	14.7 (20.8)	11.4 (22.8)	30.4 (37.2)	30.4 (37.2)	15.1 (4.7)	15.1 (4.7)	10.5 (8.8)	9.0 (10.5)	7.1 (6.5)	2.1 (9.6)	35.8 (28.5)	35.8 (28.5)	6.0 (6.1)	0.2 (8.7)
**PW6B95 QZ**	13.7 (17.5)	7.9 (20.9)	25.8 (32.4)	24.9 (33.2)	13.1 (3.4)	13.1 (3.4)	9.2 (6.8)	6.3 (9.8)	8.8 (5.3)	-2.7 (10.3)	30.4 (25.0)	30.4 (25.0)	6.3 (5.4)	-2.3 (8.2)
**SCAN TZ**	23.1 (24.2)	11.0 (31.7)	39.7 (35.9)	33.2 (42.7)	20.3 (9.0)	20.3 (9.0)	14.5 (9.2)	5.3 (16.7)	17.2 (20.7)	-3.9 (27.2)	42.1 (32.2)	40.9 (33.8)	14.0 (10.7)	-6.1 (16.9)
**SCAN QZ**	21.7 (22.1)	8.5 (29.9)	36.8 (30.6)	28.2 (39.7)	18.4 (8.6)	18.4 (8.6)	13.7 (9.6)	2.6 (17.0)	16.6 (21.8)	-6.6 (27.0)	37.5 (28.5)	36.1 (30.4)	13.6 (10.9)	-6.9 (16.4)

Table S2. MAE and ME for frequencies (in cm^{-1}) calculated for all species (overall and divided into bond types) relative to coupled-cluster results. Number in parenthesis is the standard deviation.

	Overall		Si-Si		O-H		Si-H		Si-C		Si-O		C-H	
**Method**	MAE	ME	MAE	ME	MAE	ME	MAE	ME	MAE	ME	MAE	ME	MAE	ME
**B2GP-PLYP TZ**	21.8 (20.2)	18.7 (23.1)	13.3 (12.5)	10.2 (15.1)	16.1 (13.6)	14.9 (15.0)	21.7 (14.7)	19.4 (17.7)	32.0 (31.4)	26.5 (36.3)	10.7 (11.5)	6.3 (14.5)	28.7 (14.8)	27.5 (17.0)
**B2GP-PLYP QZ**	23.0 (20.9)	20.9 (23.0)	14.9 (15.6)	13.3 (16.9)	17.3 (15.9)	16.6 (16.7)	24.9 (16.5)	22.9 (19.2)	33.3 (31.9)	29.1 (35.9)	10.9 (12.8)	8.6 (14.5)	28.9 (15.3)	27.6 (17.6)
**B3LYP TZ**	20.3 (22.1)	-3.7 (29.8)	9.0 (9.7)	-6.5 (11.5)	18.6 (14.5)	-18.6 (14.5)	17.9 (18.8)	-12.8 (22.6)	35.6 (36.1)	11.9 (49.5)	14.6 (14.4)	-9.9 (18.0)	20.6 (19.8)	-2.6 (28.5)
**B3LYP QZ**	18.6 (22.3)	-1.8 (29.1)	7.4 (9.5)	-4.4 (11.3)	16.2 (13.3)	-16.1 (13.4)	14.4 (17.2)	-8.5 (20.8)	33.6 (37.8)	14.4 (48.7)	12.8 (12.9)	-7.5 (16.5)	20.8 (19.8)	-2.6 (28.7)
**M06 TZ**	26.1 (27.7)	11.1 (36.4)	17.2 (13.5)	13.8 (17.0)	19.5 (18.5)	10.2 (25.0)	17.6 (18.5)	9.4 (23.8)	36.4 (38.7)	27.1 (45.8)	20.1 (21.5)	16.9 (24.1)	31.1 (29.3)	-10.3 (41.6)
**M06 QZ**	26.0 (25.8)	14.8 (33.6)	18.4 (15.0)	15.8 (17.7)	20.4 (18.0)	12.1 (24.5)	19.7 (18.7)	13.0 (23.8)	38.2 (33.7)	30.0 (41.2)	21.2 (21.3)	18.2 (23.9)	28.4 (28.0)	-3.2 (39.9)
**M06-2X TZ**	28.0 (29.3)	23.3 (33.2)	19.9 (18.2)	18.7 (19.4)	19.6 (17.2)	14.7 (21.7)	24.3 (22.6)	19.0 (27.2)	44.4 (39.9)	34.4 (49.0)	20.8 (19.8)	18.7 (21.8)	28.7 (29.0)	20.2 (35.6)
**M06-2X QZ**	28.5 (29.8)	24.5 (33.2)	21.2 (18.3)	20.7 (18.9)	19.4 (16.8)	14.8 (21.1)	23.9 (22.5)	18.9 (26.8)	44.6 (39.0)	35.9 (47.2)	22.7 (20.2)	21.0 (22.0)	28.2 (30.2)	20.3 (36.1)
**M11 TZ**	26.0 (30.2)	11.0 (38.4)	13.2 (13.4)	8.2 (17.0)	21.9 (13.9)	-11.0 (23.8)	19.9 (23.4)	-5.6 (30.2)	48.2 (44.9)	39.1 (53.2)	19.6 (17.7)	1.9 (26.4)	26.6 (32.1)	10.5 (40.4)
**M11 QZ**	33.4 (35.8)	4.2 (48.8)	20.1 (17.3)	6.0 (25.9)	38.3 (41.6)	-19.2 (53.5)	36.4 (40.6)	-19.8 (50.9)	51.4 (44.1)	37.4 (56.6)	28.8 (37.4)	-1.1 (47.3)	28.1 (29.9)	0.1 (41.1)
**PBE0 TZ**	21.0 (22.0)	5.7 (29.8)	10.5 (9.5)	1.4 (14.2)	16.4 (12.7)	-2.7 (20.8)	20.3 (17.4)	-11.0 (24.5)	34.5 (32.4)	24.3 (40.8)	15.1 (14.4)	1.4 (20.9)	20.3 (21.8)	5.1 (29.4)
**PBE0 QZ**	20.3 (22.2)	7.2 (29.2)	9.6 (10.3)	4.1 (13.5)	14.9 (11.4)	-1.2 (18.9)	16.9 (17.1)	-7.0 (23.0)	34.5 (32.0)	25.9 (39.4)	14.3 (13.8)	3.5 (19.6)	20.3 (21.8)	5.0 (29.5)
**PBE TZ**	50.6 (33.7)	-48.1 (37.2)	38.1 (19.6)	-38.1 (19.6)	68.4 (45.7)	-68.4 (45.7)	61.3 (35.1)	-60.6 (36.4)	37.4 (33.7)	-25.0 (43.9)	48.3 (32.1)	-46.1 (35.2)	55.2 (33.3)	-53.7 (35.7)
**PBE QZ**	48.8 (32.8)	-46.1 (36.5)	35.0 (17.6)	-35.0 (17.6)	66.4 (45.6)	-66.4 (45.6)	56.6 (32.6)	-55.8 (34.1)	36.1 (31.4)	-23.0 (42.2)	45.7 (31.2)	-43.6 (34.1)	55.8 (33.7)	-54.1 (36.4)
**PW6B95 TZ**	19.8 (24.3)	12.7 (28.7)	10.1 (10.1)	6.3 (12.8)	13.1 (11.5)	2.0 (17.4)	13.9 (18.2)	-0.3 (23.0)	34.6 (37.9)	27.1 (43.6)	12.9 (13.5)	4.2 (18.3)	22.0 (23.4)	15.4 (28.3)
**PW6B95 QZ**	20.1 (24.3)	14.4 (28.1)	11.5 (11.4)	8.9 (13.5)	13.7 (12.1)	4.7 (17.8)	14.1 (18.1)	3.7 (22.7)	35.5 (36.6)	28.9 (42.1)	13.1 (13.9)	6.5 (18.0)	22.1 (23.1)	15.4 (28.1)
**SCAN TZ**	17.7 (18.2)	-4.7 (25.0)	10.6 (8.2)	-5.0 (12.5)	16.0 (12.8)	-14.2 (14.8)	20.6 (19.8)	-14.9 (24.4)	25.9 (28.2)	3.4 (38.3)	14.4 (11.6)	-9.8 (15.7)	17.8 (22.1)	-1.9 (28.4)
**SCAN QZ**	16.5 (17.8)	-2.6 (24.2)	9.0 (8.5)	-1.7 (12.3)	14.2 (11.8)	-12.4 (13.8)	16.7 (17.9)	-9.9 (22.4)	26.7 (27.2)	6.4 (37.8)	12.9 (10.3)	-7.6 (14.7)	17.8 (21.8)	-1.8 (28.2)

Table S3. MAE and ME for zero-point energies (ZPE) (in kJ/mol) calculated for all species (overall and divided into bond types) relative to coupled-cluster results. Number in parenthesis is the standard deviation.

	Overall		Si-Si		O-H		Si-H		Si-C		Si-O		C-H	
**Method**	MAE	ME	MAE	ME	MAE	ME	MAE	ME	MAE	ME	MAE	ME	MAE	ME
**B2GP-PLYP TZ**	0.7 (0.7)	0.6 (0.7)	0.6 (0.8)	0.5 (0.9)	0.5 (0.4)	0.5 (0.4)	0.8 (0.6)	0.8 (0.6)	0.8 (0.7)	0.8 (0.8)	0.4 (0.4)	0.2 (0.5)	1.2 (0.7)	1.2 (0.7)
**B2GP-PLYP QZ**	0.7 (0.7)	0.7 (0.7)	0.7 (1.0)	0.6 (1.0)	0.5 (0.5)	0.5 (0.5)	1.0 (0.7)	1.0 (0.7)	0.8 (0.7)	0.8 (0.7)	0.4 (0.4)	0.3 (0.5)	1.2 (0.6)	1.2 (0.6)
**B3LYP TZ**	0.5 (0.5)	-0.1 (0.7)	0.4 (0.3)	-0.3 (0.4)	0.6 (0.2)	-0.6 (0.2)	0.6 (0.3)	-0.6 (0.3)	0.5 (0.8)	0.3 (0.9)	0.5 (0.3)	-0.4 (0.5)	0.5 (0.4)	-0.1 (0.7)
**B3LYP QZ**	0.4 (0.5)	-0.1 (0.6)	0.3 (0.3)	-0.2 (0.3)	0.5 (0.2)	-0.5 (0.2)	0.4 (0.2)	-0.4 (0.3)	0.5 (0.8)	0.4 (0.8)	0.4 (0.3)	-0.3 (0.4)	0.5 (0.4)	-0.1 (0.6)
**M06 TZ**	0.7 (0.6)	0.4 (0.8)	0.7 (0.5)	0.7 (0.5)	0.3 (0.3)	0.3 (0.3)	0.5 (0.3)	0.4 (0.4)	0.9 (0.8)	0.8 (0.9)	0.6 (0.6)	0.6 (0.6)	0.8 (0.7)	-0.4 (1.1)
**M06 QZ**	0.7 (0.6)	0.5 (0.8)	0.8 (0.6)	0.8 (0.6)	0.4 (0.4)	0.4 (0.4)	0.6 (0.4)	0.6 (0.4)	0.9 (0.8)	0.9 (0.9)	0.7 (0.6)	0.7 (0.6)	0.6 (0.7)	-0.1 (0.9)
**M06-2X TZ**	0.8 (0.7)	0.7 (0.7)	0.9 (0.7)	0.9 (0.7)	0.5 (0.4)	0.5 (0.4)	0.8 (0.5)	0.8 (0.5)	1.0 (0.9)	1.0 (0.9)	0.7 (0.5)	0.7 (0.5)	0.9 (0.7)	0.8 (0.8)
**M06-2X QZ**	0.8 (0.7)	0.8 (0.7)	1.0 (0.7)	1.0 (0.7)	0.5 (0.4)	0.5 (0.4)	0.8 (0.5)	0.8 (0.5)	1.0 (0.8)	1.0 (0.8)	0.8 (0.5)	0.8 (0.5)	0.9 (0.7)	0.8 (0.8)
**M11 TZ**	0.6 (0.7)	0.4 (0.9)	0.5 (0.3)	0.4 (0.4)	0.4 (0.4)	-0.3 (0.5)	0.5 (0.4)	-0.2 (0.6)	1.1 (1.0)	1.1 (1.0)	0.5 (0.4)	0.1 (0.7)	0.7 (0.7)	0.4 (0.8)
**M11 QZ**	0.8 (0.9)	0.1 (1.2)	0.7 (0.6)	0.3 (0.9)	0.7 (1.2)	-0.6 (1.3)	0.9 (1.1)	-0.9 (1.2)	1.1 (1.1)	1.1 (1.1)	0.8 (0.9)	0.0 (1.2)	0.7 (0.7)	0.0 (1.0)
**PBE0 TZ**	0.5 (0.6)	0.1 (0.7)	0.6 (0.5)	-0.1 (0.8)	0.2 (0.2)	-0.1 (0.3)	0.5 (0.5)	-0.5 (0.5)	0.7 (0.8)	0.7 (0.9)	0.4 (0.3)	0.1 (0.5)	0.4 (0.5)	0.2 (0.6)
**PBE0 QZ**	0.5 (0.5)	0.2 (0.7)	0.5 (0.4)	0.0 (0.7)	0.2 (0.1)	0.0 (0.2)	0.4 (0.4)	-0.4 (0.4)	0.7 (0.8)	0.7 (0.8)	0.4 (0.3)	0.1 (0.5)	0.4 (0.5)	0.2 (0.6)
**PBE TZ**	1.6 (1.4)	-1.6 (1.4)	2.1 (1.9)	-2.1 (1.9)	2.1 (1.0)	-2.1 (1.0)	2.6 (1.3)	-2.6 (1.3)	0.8 (0.8)	-0.7 (0.9)	1.7 (1.1)	-1.7 (1.2)	2.2 (1.5)	-2.2 (1.5)
**PBE QZ**	1.6 (1.4)	-1.5 (1.4)	1.9 (1.7)	-1.9 (1.7)	2.1 (1.0)	-2.1 (1.0)	2.4 (1.2)	-2.4 (1.2)	0.8 (0.8)	-0.6 (0.9)	1.7 (1.0)	-1.6 (1.1)	2.3 (1.5)	-2.3 (1.5)
**PW6B95 TZ**	0.5 (0.6)	0.4 (0.7)	0.4 (0.2)	0.2 (0.4)	0.1 (0.1)	0.1 (0.1)	0.2 (0.2)	-0.1 (0.3)	0.8 (0.9)	0.8 (0.9)	0.3 (0.3)	0.2 (0.4)	0.7 (0.7)	0.6 (0.7)
**PW6B95 QZ**	0.5 (0.6)	0.4 (0.6)	0.4 (0.3)	0.3 (0.4)	0.1 (0.2)	0.1 (0.2)	0.2 (0.2)	0.1 (0.3)	0.8 (0.8)	0.8 (0.8)	0.3 (0.3)	0.2 (0.4)	0.6 (0.7)	0.6 (0.7)
**SCAN TZ**	0.5 (0.4)	-0.2 (0.6)	0.6 (0.7)	-0.5 (0.8)	0.4 (0.2)	-0.4 (0.2)	0.7 (0.5)	-0.7 (0.5)	0.5 (0.5)	0.2 (0.7)	0.4 (0.3)	-0.4 (0.4)	0.3 (0.3)	-0.1 (0.4)
**SCAN QZ**	0.4 (0.4)	-0.1 (0.5)	0.5 (0.5)	-0.3 (0.6)	0.4 (0.2)	-0.4 (0.2)	0.5 (0.4)	-0.5 (0.4)	0.5 (0.5)	0.2 (0.7)	0.4 (0.3)	-0.3 (0.4)	0.3 (0.3)	-0.1 (0.4)

Table S4. Reaction energies (in kJ/mol) calculated using CCSD(T) for Si-C-O-H systems sorted by composition of reactions included in the analysis of MAE and ME (see Table S6).

Reaction composition	Reaction	Reaction energy (kJ/mol)
Si-O-H	HSiO ↔ HOSi	-32.4
	trans-HSiOH ↔ cis-HSiOH	-0.4
	cis-HSiOH ↔ H_2SiO	-4.0
	trans-HSiOH ↔ H_2SiO	-4.3
	Si + OH ↔ SiO + H	-373.1
	SiH + O ↔ Si + OH	-129.5
	Si + O_2 ↔ SiO + O	-306.2
	SiH + OH ↔ Si + H_2O	-196.3
	Si + H_2O ↔ HOSi + H	-0.2
	SiH_2 + O ↔ SiH + OH	-109.6
	HOSi + H ↔ SiO + H_2	-312.2
	SiH + O_2 ↔ HOSi + O	-129.6
	HOSi + O ↔ SiO + OH	-306.1
	SiO + OH ↔ SiO_2 + H	-6.4
	SiH_2 + O_2 ↔ H_2SiO + O	-124.5
	H_2SiO + O ↔ HOSi + OH	-114.7
	HOSi + OH ↔ SiO_2 + H_2	-318.6
	SiO_2 + H_2 ↔ SiO + H_2O	-54.2
	SiH_3 + O ↔ SiH_2 + OH	-141.0
	SiH_2 + OH ↔ SiH + H_2O	-176.3
	SiH + H_2O ↔ H_2SiO + H	-15.1
	H_2SiO + H ↔ HOSi + H_2	-120.8
	H_2SiO + OH ↔ HOSi + H_2O	-181.5
	SiH_4 + O ↔ SiH_3 + OH	-47.3
	SiH_3 + OH ↔ SiH_2 + H_2O	-207.7
	SiH_2 + H_2O ↔ H_2SiO + H_2	-130.7
	SiH_4 + O_2 ↔ H_3SiOH + O	-70.1
	H_3SiOH + O ↔ H_2SiO + H_2O	-309.4
	SiH_3SiH_3 + O ↔ SiH_3SiH + H_2O	-256.9
	SiH_2 + H_3SiOH ↔ SiH_4 + H_2SiO	-54.4
	SiH_3SiH + H_2O ↔ SiH_2 + H_3SiOH	-78.7
	SiH_3SiH_3 + OH ↔ SiH_3 + H_3SiOH	-194.6
	SiH_3SiH_3 + O_2 ↔ H_2SiO + H_3SiOH	-460.0
	SiH_3SiH + H_2O ↔ SiH_2 + H_3SiOH	-78.7
	SiH_3SiH + O ↔ SiH_3 + HOSi	-361.8
	SiH_2 + H_2SiO ↔ SiH_4 + SiO	-232.6
	SiH_3 + HOSi ↔ SiH_2 + H_2SiO	-26.3
	SiH_3SiH + OH ↔ SiH_3 + H_2SiO	-247.1
	SiH_3 + H_2SiO ↔ SiH + H_3SiOH	-7.9

	$SiH + H_3SiOH \leftrightarrow SiH_4 + HOSi$	-59.5
	$SiH_3SiH + O_2 \leftrightarrow H_2SiO + H_2SiO$	-512.5
	$H_2SiO + H_2SiO \leftrightarrow SiH_4 + SiO_2$	-47.6
	$SiH_4 + SiO_2 \leftrightarrow H_3SiOH + SiO$	-130.6
	$SiO + HOSi \leftrightarrow Si_2O_2 + H$	-106.8
	$Si_2O + OH \leftrightarrow SiO + HOSi$	-260.8
	$SiO + H_2SiO \leftrightarrow Si_2O_2 + H_2$	-227.6
	$Si_2O + H_2O \leftrightarrow SiO + H_2SiO$	-79.3
	$SiO_2 + HOSi \leftrightarrow Si_2O_2 + OH$	-100.4
	$Si_2O_2 + OH \leftrightarrow Si_2O_3 + H$	-128.7
	$SiO_2 + H_2SiO \leftrightarrow Si_2O_2 + H_2O$	-281.9
	$Si_2O_2 + H_2O \leftrightarrow Si_2O_3 + H_2$	-68.0
	$Si_2O_3 + OH \leftrightarrow Si_2O_4 + H$	-80.3
	$Si_2O_3 + H_2O \leftrightarrow Si_2O_4 + H_2$	-19.6
	$Si_2O_3 + SiH \leftrightarrow Si_2O_2 + HOSi$	-67.8
	$Si_2O_2 + HOSi \leftrightarrow Si_3O_3 + H$	-130.6
	$Si_2O_3 + SiH_2 \leftrightarrow Si_2O_2 + H_2SiO$	-62.7
	$Si_2O_2 + H_2SiO \leftrightarrow Si_3O_3 + H_2$	-251.4
	$Si_2O_3 + HOSi \leftrightarrow Si_3O_3 + OH$	-1.9
	$Si_2O_3 + H_2SiO \leftrightarrow Si_3O_3 + H_2O$	-183.4
Si-H	$SiH + H \leftrightarrow Si + H_2$	-135.6
	$SiH_2 + H \leftrightarrow SiH + H_2$	-115.6
	$SiH_3 + H \leftrightarrow SiH_2 + H_2$	-147.0
	$SiH_4 + H \leftrightarrow SiH_3 + H_2$	-53.4
	$SiH_3SiH_3 + H \leftrightarrow SiH_4 + SiH_3$	-57.6
	$SiH_3SiH + H \leftrightarrow SiH_3 + SiH_2$	-55.7
	$SiH_3 + SiH_2 \leftrightarrow SiH_4 + SiH$	-62.3
	$SiH_2 + SiH_2 \leftrightarrow SiH_4 + Si$	-50.9
	$SiH_3 + SiH \leftrightarrow SiH_2 + SiH_2$	-31.4
	$SiH_3 + Si \leftrightarrow SiH_2 + SiH$	-11.4
	$SiH + SiH \leftrightarrow SiH_2 + Si$	-20.0
	$SiH_2 + Si \leftrightarrow Si_2 + H_2$	-134.6
	$SiH + Si \leftrightarrow Si_2 + H$	-19.0
	$Si_2 + SiH \leftrightarrow Si_3 + H$	-109.5
	$Si_2 + SiH_2 \leftrightarrow Si_3 + H_2$	-225.1
	$SiH_3SiH + Si \leftrightarrow SiH_4 + Si_2$	-137.0
	$SiH_3SiH + Si_2 \leftrightarrow SiH_4 + Si_3$	-227.5
Si-O	$Si + O_2 \leftrightarrow SiO + O$	-306.2
	$SiO_2 + O \leftrightarrow SiO + O_2$	-60.5
	$Si_2O_2 + O_2 \leftrightarrow Si_2O_3 + O$	-61.8
	$Si_2O_3 + O_2 \leftrightarrow Si_2O_4 + O$	-13.4
	$Si_2 + O \leftrightarrow SiO + Si$	-483.6
	$Si_2 + O_2 \leftrightarrow Si_2O + O$	-222.9
	$Si_2O + O \leftrightarrow SiO + SiO$	-566.9

	$Si_3 + O \leftrightarrow Si + Si_2O$	-309.9
	$Si + Si_2O \leftrightarrow Si_2 + SiO$	-83.2
	$Si_3 + O_2 \leftrightarrow Si_2 + SiO_2$	-332.7
	$Si_2 + SiO_2 \leftrightarrow Si_2O_2 + Si$	-277.9
	$Si_2O_2 + Si \leftrightarrow Si_2O + SiO$	-5.5
	$Si_3 + SiO_2 \leftrightarrow Si_2O + Si_2O$	-109.7
	$Si_2O + Si_2O \leftrightarrow Si_2O_2 + Si_2$	-77.7
	$Si_2O + Si_2 \leftrightarrow Si_3 + SiO$	-173.7
	$Si_2O + Si_2O \leftrightarrow Si_2 + Si_2O_2$	-77.7
	$Si_2 + Si_2O_3 \leftrightarrow Si_2O + Si_2O_2$	-161.2
	$Si_2O + Si_2O_2 \leftrightarrow Si_3O_3 + Si$	-18.3
	$Si_3 + Si_2O_3 \leftrightarrow Si_3O_3 + Si_2$	-88.9
	$Si_2O + Si_2O_3 \leftrightarrow Si_2O_2 + Si_2O_2$	-238.9
	$Si_2 + Si_2O_4 \leftrightarrow Si_2O + Si_2O_3$	-209.6
	$Si_3 + Si_2O_4 \leftrightarrow Si_2O + Si_3O_3$	-298.5
	$Si_2O + Si_2O_3 \leftrightarrow Si_2O_2 + Si_2O_2$	-238.9
Si-O cluster	$Si + O \leftrightarrow SiO$	-798.9
	$SiO + O \leftrightarrow SiO_2$	-432.3
	$SiO + Si \leftrightarrow Si_2O$	-232.0
	$SiO + SiO \leftrightarrow Si_2O_2$	-226.6
	$SiO + SiO_2 \leftrightarrow Si_2O_3$	-348.8
	$SiO_2 + SiO_2 \leftrightarrow Si_2O_4$	-422.6
	$Si_2O_2 + SiO \leftrightarrow Si_3O_3$	-250.3
Si-C	Si_2C_2_linear $\leftrightarrow Si_2C_2$	-88.1
	$Si_2 + C \leftrightarrow SiC + Si$	-101.5
	$Si_2C + C \leftrightarrow SiC_2 + Si$	-178.6
	$Si_3 + C \leftrightarrow SiC + Si_2$	-11.0
	$SiC + Si_2 \leftrightarrow Si_2C + Si$	-327.6
	$SiC_2 + Si_2 \leftrightarrow Si_2C_2 + Si$	-79.4
	$Si_2C + SiC \leftrightarrow SiC_2 + Si_2$	-77.1
	$SiC_2 + C \leftrightarrow Si + C_3$	-80.2
	$Si_2C_2 + C \leftrightarrow Si_2 + C_3$	-0.7
	$Si_2 + C_3 \leftrightarrow SiC_2 + SiC$	-21.4
	$Si_3 + C_3 \leftrightarrow Si_2C_2 + SiC$	-10.3
	SiC_3_linear $+ Si_2 \leftrightarrow Si_3 + C_3$	-63.3
	$Si_2C_2 + SiC \leftrightarrow SiC_2 + Si_2C$	-248.2
	SiC_3_linear $+ Si_2C \leftrightarrow Si_2C_2 + SiC_2$	-150.7
	SiC_3_linear $+ SiC_2 \leftrightarrow Si_2C_2 + C_3$	-52.2
	SiC_3_linear $+ Si_3 \leftrightarrow Si_2C_2 + Si_2C$	-310.7
	$H_2SiC \leftrightarrow HCSiH$	-209.9
	$HCSiH \leftrightarrow H_2CSi$	-144.2
	$C + SiH \leftrightarrow CH + Si$	-38.3
	$CH + Si \leftrightarrow SiC + H$	-82.2
	$C + SiH_2 \leftrightarrow CH + SiH$	-18.3

$CH + SiH \leftrightarrow CH_2 + Si$	-121.6
$CH_2 + Si \leftrightarrow SiC + H_2$	-96.3
$C + SiH_3 \leftrightarrow CH + SiH_2$	-49.7
$CH + SiH_2 \leftrightarrow CH_2 + SiH$	-101.6
$CH_2 + SiH \leftrightarrow CH_3 + Si$	-161.0
$CH_3 + Si \leftrightarrow H_2CSi + H$	-60.4
$CH + SiH_3 \leftrightarrow C + SiH_4$	-44.0
$C + SiH_4 \leftrightarrow CH_2 + SiH_2$	-89.0
$CH_2 + SiH_2 \leftrightarrow CH_3 + SiH$	-141.0
$CH_3 + SiH \leftrightarrow CH_4 + Si$	-135.3
$CH_4 + Si \leftrightarrow H_2CSi + H_2$	-60.8
$CH + SiH_4 \leftrightarrow CH_2 + SiH_3$	-39.3
$CH_2 + SiH_3 \leftrightarrow CH_3 + SiH_2$	-172.4
$CH_3 + SiH_2 \leftrightarrow CH_4 + SiH$	-115.3
$CH_2 + SiH_4 \leftrightarrow CH_3 + SiH_3$	-78.8
$CH_3 + SiH_3 \leftrightarrow CH_4 + SiH_2$	-146.7
$CH_3 + SiH_4 \leftrightarrow CH_4 + SiH_3$	-53.0
$C + H_2CSi \leftrightarrow C_2H_2 + Si$	-358.1
$C_2H_2 + Si \leftrightarrow SiC_2 + H_2$	-42.0
$CH + H_2CSi \leftrightarrow CH_3 + SiC$	-21.8
$CH_3 + SiC \leftrightarrow C_2H_2 + SiH$	-298.0
$CH_2 + H_2CSi \leftrightarrow CH_4 + SiC$	-35.5
$CH_4 + SiC \leftrightarrow Si + C_2H_4$	-170.3
$Si + C_2H_4 \leftrightarrow SiH_2 + C_2H_2$	-12.4
$CH_3 + H_2CSi \leftrightarrow SiH + C_2H_4$	-44.8
$SiH + C_2H_4 \leftrightarrow SiH_3 + C_2H_2$	-1.0
$SiH_4 + C_2H_2 \leftrightarrow CH_4 + H_2CSi$	-7.2
$SiH_2 + C_2H_4 \leftrightarrow SiH_4 + C_2H_2$	-63.3
$Si + C_2H_6 \leftrightarrow SiH_2 + C_2H_4$	-51.6
$CH_2 + Si_2 \leftrightarrow Si + H_2CSi$	-202.5
$Si + H_2CSi \leftrightarrow Si_2C + H_2$	-221.5
$SiH_2 + SiC \leftrightarrow CH_2 + Si_2$	-38.4
$CH_3 + Si_2 \leftrightarrow SiH + H_2CSi$	-41.4
$SiH_3 + SiC \leftrightarrow CH_3 + Si_2$	-210.8
$SiH_4 + SiC \leftrightarrow SiH_2 + H_2CSi$	-190.0
$SiH_2 + H_2CSi \leftrightarrow CH_4 + Si_2$	-73.9
$SiH_3SiH + C \leftrightarrow SiH_4 + SiC$	-238.5
$CH + SiH_3SiH \leftrightarrow SiH_3 + H_2CSi$	-378.7
$CH_2 + SiH_3SiH \leftrightarrow SiH_4 + H_2CSi$	-339.4
$C + SiH_3SiH_3 \leftrightarrow CH_2 + SiH_3SiH$	-90.9
$C_3 + SiH_2 \leftrightarrow CH_2 + SiC_2$	-59.7
$CH_2 + SiC_2 \leftrightarrow C_2H_2 + SiC$	-54.3
$C_2H_2 + SiC \leftrightarrow H_2 + SiC_3$_linear	-47.8
$CH_2 + SiC_3$_linear $\leftrightarrow C_3 + H_2CSi$	-175.3
$Si_2 + H_2CSi \leftrightarrow Si_2C + SiH_2$	-86.8

	$Si_3 + CH_2 \leftrightarrow Si_2 + H_2CSi$	-112.0
	$SiC + H_2CSi \leftrightarrow CH_2 + Si_2C$	-125.2
	$SiH_2 + SiC_2 \leftrightarrow C_2H_2 + Si_2$	-92.7
	$C_2H_2 + Si_2 \leftrightarrow H_2 + Si_2C_2$	-121.4
	$CH_2 + Si_2C \leftrightarrow SiH_2 + SiC_2$	-38.7
	$SiC_2 + H_2CSi \leftrightarrow C_2H_2 + Si_2C$	-179.5
	$CH_2 + Si_2C_2 \leftrightarrow SiC_2 + H_2CSi$	-123.0
	SiC_3_linear + $SiH_2 \leftrightarrow CH_2 + Si_2C_2$	-111.9
	$Si_2C_2 + SiH_2 \leftrightarrow Si_2C + H_2CSi$	-84.3
	$Si_2C + H_2CSi \leftrightarrow C_2H_2 + Si_3$	-19.4
Si-C-O	$CSiO \leftrightarrow SiCO$	-270.5
	$C + SiCO \leftrightarrow SiC + CO$	-283.9
	$Si + SiCO \leftrightarrow SiC + SiO$	-10.0
	$SiC + SiO \leftrightarrow Si_2 + CO$	-172.4
	$O + SiCO \leftrightarrow SiO + CO$	-666.0
	$O_2 + SiC \leftrightarrow O + SiCO$	-296.1
	$C_3 + SiO \leftrightarrow SiC_2 + CO$	-193.7
	$Si_2 + SiCO \leftrightarrow Si_3 + CO$	-272.9
	$Si_2O + SiC \leftrightarrow Si_2 + SiCO$	-73.2
	$Si_3 + CO \leftrightarrow Si_2C + SiO$	-64.8
	$O_2 + SiCO \leftrightarrow SiO_2 + CO$	-605.6
	$SiO_2 + CO \leftrightarrow CO_2 + SiO$	-95.4
	$C_3 + SiCO \leftrightarrow SiC_3$_linear + CO	-209.6
	$SiC + SiCO \leftrightarrow SiC_2 + SiO$	-414.8
	$SiC_2 + SiO \leftrightarrow Si_2C + CO$	-95.3
	$SiC_2 + SiCO \leftrightarrow SiC_3$_linear + SiO	-15.9
	SiC_3_linear + $SiO \leftrightarrow Si_2C_2 + CO$	-245.9
	$Si_2C + SiCO \leftrightarrow Si_2C_2 + SiO$	-166.5

Table S 5. Reaction energies (in kJ/mol) of additional reactions not included in the analysis of MAE and ME as shown in Table S6. The reaction energies are calculated using CCSD(T) for Si-C-O-H systems sorted by composition.

Reaction composition	Reaction	Reaction energy (kJ/mol)
Si-O-H	$SiH_2 + O \leftrightarrow Si + H_2O$	-305.9
	$Si + H_2O \leftrightarrow SiO + H_2$	-312.4
	$SiH_2 + O \leftrightarrow SiO + H_2$	-618.3
	$SiH_2 + O \leftrightarrow HOSi + H$	-306.1
	$SiH + OH \leftrightarrow SiO + H_2$	-508.7
	$SiH + OH \leftrightarrow HOSi + H$	-196.5
	$SiH + O_2 \leftrightarrow SiO + OH$	-435.7
	$SiH + O_2 \leftrightarrow SiO_2 + H$	-442.2
	$HOSi + O \leftrightarrow SiO_2 + H$	-312.5
	$SiH_2 + O_2 \leftrightarrow HOSi + OH$	-239.2
	$SiH_2 + O_2 \leftrightarrow SiO + H_2O$	-612.0
	$H_2SiO + O \leftrightarrow SiO + H_2O$	-487.6
	$HOSi + OH \leftrightarrow SiO + H_2O$	-372.9
	$SiH_3 + O \leftrightarrow SiH + H_2O$	-317.3
	$SiH_3 + O \leftrightarrow HOSi + H_2$	-453.1
	$SiH_3 + O \leftrightarrow H_2SiO + H$	-332.3
	$SiH_2 + OH \leftrightarrow HOSi + H_2$	-312.2
	$SiH_2 + OH \leftrightarrow H_2SiO + H$	-191.4
	$SiH + H_2O \leftrightarrow HOSi + H_2$	-135.8
	$SiH_3 + O_2 \leftrightarrow H_2SiO + OH$	-265.4
	$SiH_4 + O \leftrightarrow SiH_2 + H_2O$	-255.0
	$SiH_4 + O \leftrightarrow H_2SiO + H_2$	-385.7
	$SiH_3 + OH \leftrightarrow H_2SiO + H_2$	-338.4
	$SiH_4 + O_2 \leftrightarrow H_2SiO + H_2O$	-379.5
	$SiH_3SiH_3 + O \leftrightarrow SiH_4 + H_2SiO$	-389.9
	$SiH_3SiH_3 + O \leftrightarrow SiH_2 + H_3SiOH$	-335.6
	$SiH_3SiH + O \leftrightarrow SiH_4 + SiO$	-620.6
	$SiH_3SiH + O \leftrightarrow SiH_2 + H_2SiO$	-388.1
	$SiH_3 + HOSi \leftrightarrow SiH_4 + SiO$	-258.8
	$SiH_3SiH + OH \leftrightarrow SiH_4 + HOSi$	-314.5
	$SiH_3SiH + OH \leftrightarrow SiH + H_3SiOH$	-255.0
	$SiH_3 + H_2SiO \leftrightarrow SiH_4 + HOSi$	-67.4
	$SiH_3SiH + O_2 \leftrightarrow SiH_4 + SiO_2$	-560.1
	$SiH_3SiH + O_2 \leftrightarrow H_3SiOH + SiO$	-690.7
	$H_2SiO + H_2SiO \leftrightarrow H_3SiOH + SiO$	-178.2
	$Si_2O + OH \leftrightarrow Si_2O_2 + H$	-367.6
	$SiO_2 + HOSi \leftrightarrow Si_2O_3 + H$	-229.1
	$SiO_2 + H_2SiO \leftrightarrow Si_2O_3 + H_2$	-349.9
	$Si_2O_3 + SiH \leftrightarrow Si_3O_3 + H$	-198.4

	$Si_2O_3 + SiH_2 \leftrightarrow Si_3O_3 + H_2$	-314.1
Si-H	$SiH_3SiH + H \leftrightarrow SiH_4 + SiH$	-118.0
	$SiH_3 + SiH \leftrightarrow SiH_4 + Si$	-82.3
	$SiH + SiH \leftrightarrow Si_2 + H_2$	-154.6
Si-O	$Si_2 + O_2 \leftrightarrow SiO + SiO$	-789.8
	$Si_3 + O \leftrightarrow Si_2 + SiO$	-393.1
	$Si_3 + O_2 \leftrightarrow Si_2O + SiO$	-616.1
	$Si_3 + O_2 \leftrightarrow Si_2O_2 + Si$	-610.6
	$Si_2 + SiO_2 \leftrightarrow Si_2O + SiO$	-283.4
	$Si_3 + SiO_2 \leftrightarrow Si_2O_2 + Si_2$	-187.4
	$Si_2 + Si_2O_3 \leftrightarrow Si_3O_3 + Si$	-179.4
Si-O cluster	$Si + O_2 \leftrightarrow SiO_2$	-738.4
	$Si_2 + O \leftrightarrow Si_2O$	-715.7
	$Si_2 + O_2 \leftrightarrow Si_2O_2$	-1016.4
	$Si_2O + O \leftrightarrow Si_2O_2$	-793.4
	$SiO_2 + Si \leftrightarrow Si_2O_2$	-593.2
	$Si_2O_2 + O \leftrightarrow Si_2O_3$	-554.5
	$Si_2O + O_2 \leftrightarrow Si_2O_3$	-855.2
	$Si_2O_3 + O \leftrightarrow Si_2O_4$	-506.1
	$Si_2O_3 + Si \leftrightarrow Si_3O_3$	-494.7
Si-C	$SiC + C \leftrightarrow Si + C_2$	-184.0
	$Si_2 + C_2 \leftrightarrow SiC_2 + Si$	-322.3
	$Si_2 + C_2 \leftrightarrow Si_2C + C$	-143.7
	$SiC + SiC \leftrightarrow Si_2 + C_2$	-82.4
	$SiC + SiC \leftrightarrow SiC_2 + Si$	-404.7
	$SiC + SiC \leftrightarrow Si_2C + C$	-226.1
	$Si_3 + C \leftrightarrow Si_2C + Si$	-338.7
	$Si_3 + C_2 \leftrightarrow Si_2C_2 + Si$	-311.2
	$Si_3 + C_2 \leftrightarrow SiC_2 + Si_2$	-231.8
	$Si_3 + C_2 \leftrightarrow Si_2C + SiC$	-154.7
	$Si_2C + SiC \leftrightarrow Si_2C_2 + Si$	-156.5
	$SiC + C_2 \leftrightarrow Si + C_3$	-300.9
	$SiC + C_2 \leftrightarrow SiC_2 + C$	-220.8
	$Si_2 + C_3 \leftrightarrow Si_2C + C_2$	-26.7
	$Si_2 + C_3 \leftrightarrow SiC_3\_linear + Si$	-27.2
	$Si_2C_2 + C \leftrightarrow SiC_2 + SiC$	-22.1
	$Si_2C_2 + C \leftrightarrow Si_2C + C_2$	-27.5
	$Si_2C_2 + C \leftrightarrow SiC_3\_linear + Si$	-27.9
	$SiC_2 + SiC \leftrightarrow Si_2C + C_2$	-5.4
	$SiC_2 + SiC \leftrightarrow SiC_3\_linear + Si$	-5.8
	$Si_2C + C_2 \leftrightarrow SiC_3\_linear + Si$	-0.5
	$Si_3 + C_3 \leftrightarrow SiC_2 + Si_2C$	-258.5
	$SiC_3\_linear + Si_2 \leftrightarrow Si_2C_2 + SiC$	-73.6
	$SiC_3\_linear + Si_2 \leftrightarrow SiC_2 + Si_2C$	-321.8

Si-C-H	$H_2SiC \leftrightarrow H_2CSi$	-354.1
	$C + SiH \leftrightarrow SiC + H$	-120.5
	$C + SiH_2 \leftrightarrow CH_2 + Si$	-139.9
	$C + SiH_2 \leftrightarrow SiC + H_2$	-236.2
	$CH + SiH \leftrightarrow SiC + H_2$	-217.8
	$C + SiH_3 \leftrightarrow CH_2 + SiH$	-151.3
	$C + SiH_3 \leftrightarrow CH_3 + Si$	-312.3
	$C + SiH_3 \leftrightarrow H_2CSi + H$	-372.7
	$CH + SiH_2 \leftrightarrow CH_3 + Si$	-262.6
	$CH + SiH_2 \leftrightarrow H_2CSi + H$	-323.0
	$CH_2 + SiH \leftrightarrow H_2CSi + H$	-221.4
	$C + SiH_4 \leftrightarrow CH_3 + SiH$	-230.1
	$C + SiH_4 \leftrightarrow CH_4 + Si$	-365.4
	$C + SiH_4 \leftrightarrow H_2CSi + H_2$	-426.1
	$CH + SiH_3 \leftrightarrow CH_2 + SiH_2$	-133.0
	$CH + SiH_3 \leftrightarrow CH_3 + SiH$	-274.0
	$CH + SiH_3 \leftrightarrow CH_4 + Si$	-409.3
	$CH + SiH_3 \leftrightarrow H_2CSi + H_2$	-470.1
	$CH_2 + SiH_2 \leftrightarrow CH_4 + Si$	-276.3
	$CH_2 + SiH_2 \leftrightarrow H_2CSi + H_2$	-337.1
	$CH_3 + SiH \leftrightarrow H_2CSi + H_2$	-196.1
	$CH + SiH_4 \leftrightarrow CH_3 + SiH_2$	-211.7
	$CH + SiH_4 \leftrightarrow CH_4 + SiH$	-327.1
	$CH_2 + SiH_3 \leftrightarrow CH_4 + SiH$	-287.7
	$CH_2 + SiH_4 \leftrightarrow CH_4 + SiH_2$	-225.5
	$CH_2 + SiC \leftrightarrow C + H_2CSi$	-100.9
	$SiH_2 + C_2 \leftrightarrow C + H_2CSi$	-56.8
	$C + H_2CSi \leftrightarrow SiC_2 + H_2$	-400.1
	$CH_2 + SiC \leftrightarrow SiH_2 + C_2$	-44.1
	$CH_2 + SiC \leftrightarrow SiC_2 + H_2$	-501.0
	$SiH_2 + C_2 \leftrightarrow SiC_2 + H_2$	-456.9
	$SiH_2 + C_2 \leftrightarrow C_2H_2 + Si$	-415.0
	$SiH_3 + C_2 \leftrightarrow CH + H_2CSi$	-106.6
	$CH + H_2CSi \leftrightarrow C_2H_2 + SiH$	-319.8
	$SiH_3 + C_2 \leftrightarrow CH_3 + SiC$	-128.3
	$SiH_3 + C_2 \leftrightarrow C_2H_2 + SiH$	-426.4
	$SiH_4 + C_2 \leftrightarrow CH_2 + H_2CSi$	-145.9
	$CH_2 + H_2CSi \leftrightarrow SiH_2 + C_2H_2$	-218.2
	$CH_2 + H_2CSi \leftrightarrow Si + C_2H_4$	-205.8
	$SiH_4 + C_2 \leftrightarrow CH_4 + SiC$	-181.4
	$SiH_4 + C_2 \leftrightarrow SiH_2 + C_2H_2$	-364.1
	$SiH_4 + C_2 \leftrightarrow Si + C_2H_4$	-351.7
	$CH_4 + SiC \leftrightarrow SiH_2 + C_2H_2$	-182.7
	$CH_3 + H_2CSi \leftrightarrow SiH_3 + C_2H_2$	-45.8
	$SiH_2 + C_2H_4 \leftrightarrow CH_4 + H_2CSi$	-70.5

	$Si + C_2H_6 \leftrightarrow CH_4 + H_2CSi$	-122.1
	$Si + C_2H_6 \leftrightarrow SiH_4 + C_2H_2$	-114.9
	$SiH_2 + SiC \leftrightarrow Si + H_2CSi$	-240.8
	$SiH_2 + SiC \leftrightarrow Si_2C + H_2$	-462.3
	$CH_2 + Si_2 \leftrightarrow Si_2C + H_2$	-423.9
	$SiH_3 + SiC \leftrightarrow SiH + H_2CSi$	-252.2
	$SiH_3SiH + C \leftrightarrow SiH_2 + H_2CSi$	-428.5
	$SiH_3SiH + C \leftrightarrow CH_4 + Si_2$	-502.3
	$SiH_4 + SiC \leftrightarrow CH_4 + Si_2$	-263.8
	$C + SiH_3SiH_3 \leftrightarrow SiH_4 + H_2CSi$	-430.3
	$C_2 + H_2CSi \leftrightarrow C_3 + SiH_2$	-60.1
	$C_2 + H_2CSi \leftrightarrow C_2H_2 + SiC$	-174.1
	$C_2 + H_2CSi \leftrightarrow CH_2 + SiC_2$	-119.8
	$C_2 + H_2CSi \leftrightarrow H_2 + SiC_3$_linear	-221.9
	$C_3 + SiH_2 \leftrightarrow C_2H_2 + SiC$	-114.0
	$C_3 + SiH_2 \leftrightarrow H_2 + SiC_3$_linear	-161.8
	$CH_2 + SiC_2 \leftrightarrow H_2 + SiC_3$_linear	-102.1
	$Si_3 + CH_2 \leftrightarrow Si_2C + SiH_2$	-198.8
	$SiC + H_2CSi \leftrightarrow SiC_2 + SiH_2$	-163.9
	$SiC + H_2CSi \leftrightarrow C_2H_2 + Si_2$	-256.6
	$SiC + H_2CSi \leftrightarrow SiH_2 + SiC_2$	-163.9
	$SiC + H_2CSi \leftrightarrow H_2 + Si_2C_2$	-378.0
	$CH_2 + Si_2C \leftrightarrow C_2H_2 + Si_2$	-131.4
	$SiH_2 + SiC_2 \leftrightarrow H_2 + Si_2C_2$	-214.0
	$CH_2 + Si_2C \leftrightarrow H_2 + Si_2C_2$	-252.8
	SiC_3_linear $+ SiH_2 \leftrightarrow SiC_2 + H_2CSi$	-235.0
	SiC_3_linear $+ SiH_2 \leftrightarrow C_2H_2 + Si_2C$	-414.5
	$CH_2 + Si_2C_2 \leftrightarrow C_2H_2 + Si_2C$	-302.5
	$Si_2C_2 + SiH_2 \leftrightarrow C_2H_2 + Si_3$	-103.8
Si-C-O	$C + SiCO \leftrightarrow C_2 + SiO$	-194.0
	$C_2 + SiO \leftrightarrow SiC + CO$	-89.9
	$Si + SiCO \leftrightarrow Si_2 + CO$	-182.4
	$O_2 + SiC \leftrightarrow SiO + CO$	-962.2
	$C_2 + SiCO \leftrightarrow SiC_2 + CO$	-504.7
	$C_2 + SiCO \leftrightarrow C_3 + SiO$	-311.0
	$Si_2 + SiCO \leftrightarrow Si_2C + SiO$	-337.7
	$Si_2O + SiC \leftrightarrow Si_3 + CO$	-346.1
	$Si_2O + SiC \leftrightarrow Si_2C + SiO$	-410.9
	$O_2 + SiCO \leftrightarrow CO_2 + SiO$	-701.0
	$SiC + SiCO \leftrightarrow Si_2C + CO$	-510.0
	$SiC_2 + SiCO \leftrightarrow Si_2C_2 + CO$	-261.8

Table S6. MAE and ME for reaction energies for the Si-C-O-H system (overall and sorted according to composition of reaction system) for all the reactions listed in

	Overall		Si-C		Si-C-H		Si-C-O		Si-H		Si-O		Si-O-H		Si-O cluster	
**Method**	**MAE**	**ME**	**MAE**	**ME**	**MAE**	**ME**	**MAE**	**ME**	**MAE**	**ME**	**MAE**	**ME**	**MAE**	**ME**	**MAE**	**ME**
**B2GP-PLYP**	7.4 (5.8)	1.5 (9.2)	8.2 (6.2)	0.8 (10.3)	7.2 (5.7)	1.6 (9.0)	7.8 (6.9)	3.3 (9.9)	8.4 (6.6)	2.0 (10.5)	7.3 (6.1)	2.7 (9.2)	7.1 (5.9)	1.8 (9.1)	7.9 (5.7)	5.7 (8.0)
**B3LYP**	13.8 (12.1)	6.9 (17.1)	14.9 (10.9)	6.3 (17.5)	13.1 (11.5)	6.6 (16.2)	18.8 (14.8)	10.6 (21.6)	16.8 (14.1)	7.4 (20.7)	17.3 (13.6)	8.2 (20.5)	13.1 (11.0)	6.6 (15.9)	15.6 (11.7)	10.1 (16.8)
**M06**	12.3 (10.1)	3.8 (15.5)	13.1 (10.3)	0.3 (16.7)	13.3 (10.7)	3.2 (16.8)	13.8 (11.6)	3.4 (17.8)	12.1 (9.4)	4.1 (14.9)	12.6 (10.7)	3.0 (16.3)	9.8 (7.1)	2.3 (11.9)	11.7 (8.1)	0.8 (14.3)
**M06-2X**	15.0 (11.6)	-1.1 (19.0)	15.9 (12.9)	-3.8 (20.2)	14.4 (10.7)	-0.3 (18.0)	17.7 (13.4)	1.2 (22.3)	14.4 (12.4)	1.1 (19.1)	17.7 (12.9)	-0.1 (22.0)	13.7 (10.2)	-2.3 (17.0)	17.5 (9.7)	-2.5 (20.1)
**M11**	14.7 (12.9)	0.2 (19.6)	18.4 (15.4)	-4.2 (23.8)	13.7 (12.1)	0.6 (18.2)	18.7 (14.5)	2.9 (23.6)	15.1 (11.6)	1.8 (19.0)	16.9 (13.9)	1.5 (21.9)	12.8 (11.2)	-0.2 (17.0)	13.8 (11.7)	-1.4 (18.2)
**PBE0**	17.8 (16.8)	8.4 (23.0)	16.4 (12.9)	3.2 (20.8)	17.8 (16.2)	7.2 (23.0)	22.4 (23.3)	12.6 (29.9)	20.3 (20.7)	9.5 (27.5)	21.0 (20.3)	10.6 (27.3)	16.9 (14.4)	7.8 (20.9)	19.2 (17.0)	8.8 (24.3)
**PBE**	24.0 (22.6)	7.9 (32.1)	27.4 (23.3)	6.5 (35.6)	22.0 (21.8)	5.4 (30.5)	28.8 (25.2)	12.3 (36.4)	23.9 (24.8)	6.9 (33.8)	25.8 (24.3)	9.5 (34.3)	22.4 (21.0)	6.1 (30.2)	26.9 (24.7)	11.8 (34.8)
**PW6B95**	9.9 (10.6)	4.1 (14.0)	9.9 (8.3)	0.7 (13.0)	9.2 (10.6)	3.8 (13.6)	14.2 (14.2)	6.8 (18.9)	11.8 (13.6)	5.2 (17.3)	13.3 (12.9)	5.5 (17.7)	9.5 (9.1)	3.7 (12.7)	12.5 (11.2)	5.4 (16.0)
**SCAN**	22.2 (21.6)	11.0 (29.0)	22.6 (22.4)	10.4 (30.2)	20.9 (20.3)	8.3 (28.0)	22.8 (23.7)	13.3 (30.2)	20.0 (19.6)	8.6 (26.7)	21.0 (20.6)	9.5 (27.9)	18.6 (15.5)	8.0 (22.9)	17.8 (14.3)	7.1 (21.9)

Table S4. Number in parenthesis is the standard deviation. Note that reactions including C_2 are omitted.

Notes on the calculation of C_2:

C_2 is a notoriously difficult case to calculate correctly due to a large number of electronic states that are very close in energy [Faraday Discuss., 2019, 217, 514]. A large number of calculations were performed in the search of the lowest lying electronic state, with only partial success. It is known in literature that molecular C_2 is particularly challenging, and has been described as being a prototypical example of multireference character in electronic structure. The open shell singlet was obtained by providing non-symmetric (from the triplet state) orbitals as input. While this is a technicality, it is worth mentioning. However, the open shell singlet was consistently higher in energy than the triplet with an energy difference between 15 kJ/mol (SCAN) and 75 kJ/mol (M06). Interestingly, the Minnesota functionals performed significantly worse on C_2 compared to the other functionals. M06-2x and M11 had a Δ(triplet→singlet) of 42 and 31 kJ/mol, respectively, while B3LYP, PBE0 and PBE all had an energy difference of around 24 kJ/mol. In all cases, the open shell singlet was higher in energy compared to the triplet, which is not in agreement with experiments or the coupled cluster results. A detailed investigation of the electronic states showed that the correct electronic ground state was never obtained.

Optimized structures (in xyz-format) of the molecules from Table 2 as calculated using CCSD(T). Basis set used is aug-cc-pV(Q+d)Z except for SiH_3H_3 and SiH_3SiH where aug-cc-pV(T+d)Z was used.

C_2H_2 (spin multiplicity: 1)

```
4
C2H2
C   0.00000000   0.00000000    0.60342405
C   0.00000000   0.00000000   -0.60342405
H   0.00000000   0.00000000    1.66697767
H   0.00000000   0.00000000   -1.66697767
```

C_2H_4 (spin multiplicity: 1)

```
6
C2H4
C   -0.66736630   0.00000000   0.00000000
C   0.66736630    0.00000000   0.00000000
H   1.23202218    0.92372350   0.00000000
H   1.23202218   -0.92372350   0.00000000
H   -1.23202218   0.92372350   0.00000000
H   -1.23202218  -0.92372350   0.00000000
```

C_2H_6 (spin multiplicity: 1)

```
8
C2H6
C   0.00000000    0.76349446    0.00000000
C   0.00000000   -0.76349446    0.00000000
H   -0.50866688  -1.15791326   -0.88103688
H   -0.50866688  -1.15791326    0.88103688
H   1.01733376   -1.15791326    0.00000000
H   0.50866688    1.15791326    0.88103688
H   -1.01733376   1.15791326    0.00000000
H   0.50866688    1.15791326   -0.88103688
```

C_2 (spin multiplicity: 1)

```
2
C2
C   0.00000000   0.00000000    0.62301783
C   0.00000000   0.00000000   -0.62301783
```

C_2 (spin multiplicity: 3)

```
2
C2 3Pi
C   0.00000000   0.00000000    0.65766308
C   0.00000000   0.00000000   -0.65766308
```

C_3 (spin multiplicity: 1)
3
C3

C	0.00000000	0.00000000	1.29371963
C	0.00000000	0.00000000	0.00000000
C	0.00000000	0.00000000	-1.29371963

CH_2 (spin multiplicity: 3)
3
CH2

H	0.00000000	-0.99075773	0.36272671
C	0.00000000	0.00000000	-0.06092751
H	0.00000000	0.99075773	0.36272671

CH_3 (spin multiplicity: 2)
4
CH3

C	0.00000000	0.00000000	0.00000000
H	-0.93360162	0.00000000	0.53901515
H	0.00000000	0.00000000	-1.07803030
H	0.93360162	0.00000000	0.53901515

CH_4 (spin multiplicity: 1)
5
CH4

C	0.00000000	0.00000000	0.00000000
H	0.62828769	-0.62828769	0.62828769
H	-0.62828769	0.62828769	0.62828769
H	-0.62828769	-0.62828769	-0.62828769
H	0.62828769	0.62828769	-0.62828769

CH (spin multiplicity: 2)
2
CH

C	0.00000000	0.00000000	0.08679596
H	0.00000000	0.00000000	-1.03346467

cis-HSiOH (spin multiplicity: 1)
4
cis-HSiOH

H	-0.83301137	1.46866124	0.00000000
Si	-0.61745207	-0.05195526	0.00000000
O	1.03842951	-0.05164117	0.00000000

H	1.49266973	0.79318440	0.00000000

CO_2 (spin multiplicity: 1)
3
CO2

O	0.00000000	0.00000000	1.16309645
C	0.00000000	0.00000000	0.00000000
O	0.00000000	0.00000000	-1.16309645

CO (spin multiplicity: 1)
2
CO

C	0.00000000	0.00000000	0.64665556
O	0.00000000	0.00000000	-0.48514587

CSi_2 (spin multiplicity: 1)
3
CSi2

Si	0.00000000	-1.43357783	0.16157971
C	0.00000000	0.00000000	-0.75341728
Si	0.00000000	1.43357783	0.16157971

CSiO (spin multiplicity: 3)
3
CSiO

C	0.00000000	0.00000000	1.88777284
Si	0.00000000	0.00000000	0.03704720
O	0.00000000	0.00000000	-1.48107955

H_2CO (spin multiplicity: 1)
4
H2CO

O	0.00000000	0.00000000	-0.60284499
C	0.00000000	0.00000000	0.60470778
H	0.00000000	0.93787965	1.18371778
H	0.00000000	-0.93787965	1.18371778

H_2CSi (spin multiplicity: 1)
4
H2CSi

Si	0.00000000	0.00000000	-0.60125013
C	0.00000000	0.00000000	1.11435743
H	0.00000000	0.91168660	1.71103213

H 0.00000000 -0.91168660 1.71103213

H_2O (spin multiplicity: 1)
3
H2O
H 0.00000000 -0.75754434 0.52215008
O 0.00000000 0.00000000 -0.06580040
H 0.00000000 0.75754434 0.52215008

H_2SiC (spin multiplicity: 1)
4
H2SiC
C 0.00000000 0.00000000 -1.31436656
Si 0.00000000 0.00000000 0.47498574
H 0.00000000 1.26705200 1.23223641
H 0.00000000 -1.26705200 1.23223641

H_2SiO (spin multiplicity: 1)
4
H2SiO
O 0.00000000 0.00000000 -1.02955909
Si 0.00000000 0.00000000 0.49342738
H 0.00000000 1.22477902 1.32122524
H 0.00000000 -1.22477902 1.32122524

H_2 (spin multiplicity: 1)
2
H2
H 0.00000000 0.00000000 0.37099350
H 0.00000000 0.00000000 -0.37099350

H_3SiOH (spin multiplicity: 1)
6
H3SiOH
H -1.01726556 1.39686262 0.00000000
Si -0.56310112 -0.00549056 0.00000000
O 1.08623495 0.05879845 0.00000000
H 1.57014733 -0.76630215 0.00000000
H -1.08034845 -0.70565909 1.19911803
H -1.08034845 -0.70565909 -1.19911803

HCO (spin multiplicity: 2)
3

HCO
H -1.35316025 0.74482154 0.00000000
C -0.62057456 -0.10167460 0.00000000
O 0.55084029 0.02934967 0.00000000

HCSiH (spin multiplicity: 1)
4
HCSiH
H -2.12536174 0.33603627 0.00000000
C -1.13779055 -0.09656270 0.00000000
Si 0.51191028 0.06761368 0.00000000
H 1.46235976 -1.06321648 0.00000000

HOSi (spin multiplicity: 2)
3
HOSi
H -1.51059780 0.76133110 0.00000000
O -1.01622758 -0.06232943 0.00000000
Si 0.63541259 0.00820910 0.00000000

HSiO (spin multiplicity: 2)
3
HSiO
H -1.36835129 1.19678433 0.00000000
Si -0.52395585 -0.06695810 0.00000000
O 1.00267702 0.04170906 0.00000000

O_2 (spin multiplicity: 3)
2
O2
O 0.00000000 0.00000000 0.60408278
O 0.00000000 0.00000000 -0.60408278

OH (spin multiplicity: 2)
2
OH
H 0.00000000 0.00000000 0.91309404
O 0.00000000 0.00000000 -0.05753322

Si_2C_2 (spin multiplicity: 1)
4
Si2C2
Si -1.68008023 0.00000000 0.00000000

C 0.00000000 0.72777955 0.00000000
C 0.00000000 -0.72777955 0.00000000
Si 1.68008023 0.00000000 0.00000000

Si_2O_2 (spin multiplicity: 1)
4
Si2O2
Si 1.22422637 0.00000000 0.00000000
O 0.00000000 1.17197568 0.00000000
Si -1.22422637 0.00000000 0.00000000
O 0.00000000 -1.17197568 0.00000000

Si_2O_3 (spin multiplicity: 1)
5
Si2O3
O 0.00000000 -1.17133767 0.39265519
Si 0.00000000 0.00000000 1.63234117
O 0.00000000 1.17133767 0.39265519
Si 0.00000000 0.00000000 -0.77539377
O 0.00000000 0.00000000 -2.28420898

Si_2O_4 (spin multiplicity: 1)
6
Si2O4
O 0.00000000 -1.17003167 0.00000000
Si 1.18367230 0.00000000 0.00000000
O 0.00000000 1.17003167 0.00000000
Si -1.18367230 0.00000000 0.00000000
O -2.68999293 0.00000000 0.00000000
O 2.68999293 0.00000000 0.00000000

Si_2O (spin multiplicity: 1)
3
Si2O
Si 0.00000000 -1.16558170 0.27715059
O 0.00000000 0.00000000 -0.96953589
Si 0.00000000 1.16558170 0.27715059

Si_2 (spin multiplicity: 3)
2
Si2
Si 0.00000000 0.00000000 1.12700236
Si 0.00000000 0.00000000 -1.12700236

Si_3O_3 (spin multiplicity: 1)
6
Si3O3
O 0.00000000 -1.29542219 -0.74791235
Si 0.00000000 -1.55462630 0.89756391
O 0.00000000 0.00000000 1.49582470
Si 0.00000000 1.55462630 0.89756391
O 0.00000000 1.29542219 -0.74791235
Si 0.00000000 0.00000000 -1.79512782

Si_3 (spin multiplicity: 1)
3
Si3
Si 0.00000000 -1.37389675 0.56581762
Si 0.00000000 0.00000000 -1.13163525
Si 0.00000000 1.37389675 0.56581762

Si_2C_2_linear (spin multiplicity: 1)
4
Si2C2-linear
Si 0.00000000 0.00000000 2.39122998
C 0.00000000 0.00000000 0.64060211
C 0.00000000 0.00000000 -0.64060211
Si 0.00000000 0.00000000 -2.39122998

SiC_2 (spin multiplicity: 1)
3
SiC2
C 0.00000000 0.63739587 0.92928376
Si 0.00000000 0.00000000 -0.79718584
C 0.00000000 -0.63739587 0.92928376

SiC_3_linear (spin multiplicity: 1)
4
SiC3-linear
Si 0.00000000 0.00000000 1.71355349
C 0.00000000 0.00000000 -0.02953622
C 0.00000000 0.00000000 -1.32634648
C 0.00000000 0.00000000 -2.63911404

SiCO (spin multiplicity: 3)
3
SiCO

Si	0.00000000	0.00000000	1.24201093
C	0.00000000	0.00000000	-0.57994098
O	0.00000000	0.00000000	-1.73732453

SiC (spin multiplicity: 3)
2
SiC

Si	0.00000000	0.00000000	0.51605885
C	0.00000000	0.00000000	-1.20314507

SiH_2 (spin multiplicity: 1)
3
SiH2

H	0.00000000	-1.09391834	0.98038058
Si	0.00000000	0.00000000	-0.07063335
H	0.00000000	1.09391834	0.98038058

SiH_3SiH_3 (spin multiplicity: 1)
8
SiH3SiH3

Si	0.00000000	1.17396009	0.00000000
Si	0.00000000	-1.17396009	0.00000000
H	-0.69584233	-1.68787981	-1.20523426
H	-0.69584233	-1.68787981	1.20523426
H	1.39168465	-1.68787981	0.00000000
H	0.69584233	1.68787981	1.20523426
H	-1.39168465	1.68787981	0.00000000
H	0.69584233	1.68787981	-1.20523426

SiH_3SiH (spin multiplicity: 1)
6
SiH3SiH

Si	-1.13093898	-0.00171175	0.00000000
Si	1.26450825	-0.05529489	0.00000000
H	1.26848191	1.46643155	0.00000000
H	-1.76465375	-1.34410345	0.00000000
H	-1.60583599	0.73007972	-1.20657028
H	-1.60583599	0.73007972	1.20657028

SiH_3 (spin multiplicity: 2)
4
SiH3

Si	0.04373491	0.00000000	0.00000000
H	-0.40468943	0.70519454	-1.22143277

H	-0.40468943	-1.41038908	0.00000000
H	-0.40468943	0.70519454	1.22143277

SiH_4 (spin multiplicity: 1)
5
SiH4

Si	0.00000000	0.00000000	0.00000000
H	0.85393742	-0.85393742	0.85393742
H	-0.85393742	0.85393742	0.85393742
H	-0.85393742	-0.85393742	-0.85393742
H	0.85393742	0.85393742	-0.8539374

SiH (spin multiplicity: 2)
2
SiH

Si	0.00000000	0.00000000	0.05294210
H	0.00000000	0.00000000	-1.46965716

SiO_2 (spin multiplicity: 1)
3
SiO2

O	0.00000000	0.00000000	1.51369447
Si	0.00000000	0.00000000	0.00000000
O	0.00000000	0.00000000	-1.51369447

SiO (spin multiplicity: 1)
2
SiO

Si	0.00000000	0.00000000	0.55207714
O	0.00000000	0.00000000	-0.96564579

trans-HSiOH (spin multiplicity: 1)
4
trans-HSiOH

H	-0.87076451	1.43156611	0.00000000
Si	-0.61609115	-0.06728803	0.00000000
O	1.03760267	0.07553703	0.00000000
H	1.50576662	-0.76249782	0.00000000
